# Phosphorylation-Induced Ubiquitination and Degradation of PXR through CDK2-TRIM21 Axis

**DOI:** 10.3390/cells11020264

**Published:** 2022-01-13

**Authors:** Mengyao Qin, Yu Xin, Yong Bian, Xuan Yang, Tao Xi, Jing Xiong

**Affiliations:** 1 Department of Pharmacology, School of Pharmacy, China Pharmaceutical University, Nanjing 210009, China; 3319071190@stu.cpu.edu.cn (M.Q.); YU.XIN@cttq.com (Y.X.); yangxuan_516@163.com (X.Y.); 2 Laboratory Animal Center, Nanjing University of Chinese Medicine, Nanjing 210023, China; 150945@njucm.edu.cn; 3 Research Center of Biotechnology, School of Life Science and Technology, China Pharmaceutical University, Nanjing 210009, China; xitao18@hotmail.com

**Keywords:** tripartite motif containing 21, cyclin dependent kinase 2, pregnane X receptor, drug-metabolizing enzymes and transporters, ubiquitination, dinaciclib

## Abstract

Pregnane X receptor (PXR) is a member of the nuclear receptor superfamily that is activated by a variety of endogenous metabolites or xenobiotics. Its downstream target genes are involved in metabolism, inflammation and processes closely related to cancer. However, the stability regulation of PXR protein resulting from post-translational modification is still largely undefined. In the present study, primary mouse hepatocytes, hepatoma HepG2 cells and HEK 293T cells were used to investigate gene expression and protein interactions. The role of kinases was evaluated by RNA interference and overexpression constructs with or without PXR phosphorylation site mutations. The activity of CYP3A4 and P-gp was determined by enzymatic and substrate accumulation assays. It was found that E3 ubiquitin ligase TRIM21 mediates the ubiquitination and degradation of PXR and plays an important role in regulating the activity of PXR. On this basis, PXR phosphorylation-associated kinases were evaluated regarding regulation of the stability of PXR. We found cyclin dependent kinase 2 (CDK2) exclusively phosphorylates PXR at Ser350, promotes its disassociation with Hsp90/DNAJC7, and leads to subsequent TRIM21-mediated PXR ubiquitination and degradation. As well-known CDK inhibitors, dinaciclib and kenpaullone stabilize PXR and result in elevated expression and activity of PXR-targeted DMETs, including carboxylesterases, CYP3A4 and P-gp. The suppressed degradation of PXR by CDK2 inhibitors denotes dinaciclib-induced promotion of PXR-targeted genes. The findings of CDK2-mediated PXR degradation indicate a wide range of potential drug–drug interactions during clinical cancer therapy using CDK inhibitors and imply an alternative direction for the development of novel PXR antagonists.

## 1. Introduction

Nuclear receptors (NRs), including constitutive androstane receptor (CAR, NRII3) and pregnane X receptor (PXR, NRII2), are key modulators governing the inductive expression of drug-metabolizing enzymes and transporters (DMETs) [1]. As one of the most important NRs, PXR is crucial for the biotransformation of many therapeutic agents [2]. PXR is predominantly expressed in liver tissue, with lower detection in intestine, colon, kidney, brain, and mammary tissue [3]. Representative target genes of PXR are the DMETs, including major hydrolase carboxylesterases, CYP3A family and drug transporters such as P-glycoprotein (P-gp), encoded by MDR1 [4].

Post-translational modification of PXR, including phosphorylation [5], acetylation [6], SUMOylation [7], poly (ADP-ribosylation) [8] and ubiquitination [3], modulates PXR properties such as subcellular localization, dimerization, and co-modulator interaction, as well as protein stability [9,10]. Phosphorylation of PXR has been reported to regulate the transcriptional activity of PXR [11,12] and, therefore, control the target DMET genes, leading to the alteration of drug metabolism. Phosphorylation of PXR mainly occurs on the serine and threonine residues that are mainly associated with the negative regulation of PXR activity. Many studies in recent years indicate that the protein kinases regulating PXR include PKC (protein kinase C), Akt (protein kinase B), CaMKII (Ca^2+^/calmodulin-dependent protein kinase II), CK2 (casein kinase 2) and PKA (cyclic-AMP dependent protein kinase A), of which cyclin-dependent kinases (CDKs) are typical ones. The application of CDK or MAPK kinase inhibitors to hepatoma/hepatocarcinoma cells increases PXR-mediated regulation [13,14]. However, the mechanisms of PXR phosphorylation leading to the inhibition of transcriptional activity remain unclear till now. It is only partially known that phosphorylation of PXR alters its subcellular localization or abrogates the recruitment of co-activators [15,16].

Protein degradation is an important and versatile housekeeping function essential for cell viability in eukaryotic cells. The ATP/ubiquitin (Ub)-dependent 26S proteasomal system (Ub/26S) enables intracellular protein degradation under a highly specific control. E3 enzymes, ubiquitin-protein ligases, conjugate ubiquitin to the specific protein substrate. The ubiquitinated protein is, therefore, recognized by the proteasome for its eventual degradation [17]. TRIM21 (tripartite motif containing 21) is a member of the TRIM protein family of RING E3 ubiquitin ligases. TRIM21 has E3 ligase activity and functions in the process of ubiquitination. Previous study reports that interaction between TRIM21 and PXR exists, which was identified by Flag-PXR based immunoaffinity chromatography without further elucidation regarding the effect of TRIM21 on the degradation of PXR [18].

CDKs are serine/threonine kinases that regulate cell cycle progression through combination with various cyclins in specific phases of the cell cycle [19]. Cyclin-CDK complexes drive the cell cycle through its respective phases in response to different signals, such as mitogenic signals [20]. Dysregulated cell-cycle machinery has been identified as an essential driver of malignant behavior and consecutively as a candidate therapeutic target [21]; therefore, CDK inhibitors represent a promising therapeutic approach in many malignancies [22,23]. Types of cyclin-dependent kinases include CDK1, CDK2, CDK3, CDK4, CDK5, CDK6, CDK7, CDK8, CDK9, CDK10, and CDK11 [19,24]. The treatment of CDK2 inhibitors results in cell cycle arrest and apoptosis induction [25]. Dinaciclib is a potent and specific CDK inhibitor that represses CDK1, CDK2, CDK5 and CDK9, and is metabolized via CYP3A4. Preclinical studies have shown that dinaciclib arrests cell cycle progression, induces apoptosis, and inhibits tumor growth in multiple types of cancer [26,27]. Encouraging Phase I and II results provide a rationale for the use of dinaciclib alone or in combination with immunotherapies in chronic lymphocytic leukemia and lymphoma [23]. With the increasing burden of patients with multiple disease states, drug therapy has been accompanied by more complexity. The complex therapeutic regimens increase the risk of drug–drug interactions (DDIs) to a great extent [28,29]. The changes in the expression and activity of DMETs are the fundamental biological basis for pharmacokinetic DDIs [30].

On the above basis, it is of great essence to demonstrate mechanisms of phosphorylation on PXR stabilization, in order to provide information relevant to rational drug use and to alleviate adverse drug reactions in cancer therapy. It is hypothesized that the suppressed degradation of PXR induced by CDK inhibitors is translated into the positive regulation of DMET expression and activity. Our findings could also contribute to strategies for better DDI control in cancer therapy by suggesting potential targets, including E3 ligase TRIM21.

## 2. Materials and Methods

### 2.1. Cell Culture

The human hepatoma cell line HepG2 and the HEK 293T cell line were purchased from the cell bank of Shanghai Institute of Biochemistry and Cell Biology, Chinese Academy of Sciences. Each cell line was confirmed to be mycoplasma-free, and the cells were passaged no more than 25 times after thawing. Cells were cultured in DMEM supplemented with 10% FBS, 100 U/mL penicillin and 100 U/mL streptomycin in a humidified environment with 5% CO_2_ at 37 °C.

### 2.2. Plasmids and siRNA

The plasmids encoding full-length pcDNA3.1-EGFP-PXR-Flag, hPXR-S350D and hPXR-S350A plasmids were purchased from Obio Technology (Shanghai, China). Full-length TRIM21 was cloned from pGEM-hTRIM21 clone into pcDNA-3.1. The pGEM-hTRIM21 cDNA clone was purchased from Sino Biological (Beijing, China). All constructs were verified by sequencing. HA-CDK5 and HA-CDK2 were purchased from PPL (Nanjing, China). pcDNA3-HA-Ub was from Addgene. For silencing CDK2/5 expression, siCDK2, siCDK5 and control siRNA were purchased from Gene Pharma (Shanghai, China). For silencing TRIM21 expression, siTRIM21 and control siRNA were purchased from Tsingke (Nanjing, China). Plasmid DNA was transfected using X-treme GENE HP DNA Transfection Reagent (Roche, Basel, Switzerland) according to the manufacturer’s protocol. siRNA transfections were performed with a final concentration of 100 pmol/L using Lipofectamine 2000 transfection reagent (Invitrogen, Waltham, MA, USA) according to the manufacturer’s recommendation.

### 2.3. Cell Viability Assay

Cell viability assay was determined by the MTT method. Cells were treated with indicated concentrations of dinaciclib for 24 h, then added with 20 μL of MTT in 5 mg/mL and incubated at 37 °C for 4 h. Subsequently, the culture medium was discarded, and DMSO (150 μL) was added into each well to dissolve the formazan crystals. The absorbance was then evaluated at 570 nm.

### 2.4. Western Blot Analysis

The cell lysates (30 μg) of HepG2 were resolved by 8% SDS-polyacrylamide gel and transferred electrophoretically onto polyvinylidene difluoride membranes (Merck Millipore, Boston, MA, USA). The membranes were blocked with 5% non-fat milk overnight at 4 °C, then cut according to the molecular weight of protein marker, and incubated with different primary antibodies at 4 °C overnight against CES1 (1:5000), CES2 (1:5000), P-gp (1:5000) and PXR (1:3000). The immune complexes were then incubated with horseradish peroxidase-conjugated secondary antibody for 50 min and visualized with enhanced chemiluminescence detection system.

Phosphorylated PXR was detected using phos-tag reagent (Apexbio, Houston, TX, USA), which can specifically bind to the phosphorylated proteins, and then detected by anti-PXR antibody. In brief, phos-tag gels were prepared as 10% polyacrylamide SDS-PAGE gel with the addition of 5 mM phos-tag reagent and 10 mM MnCl_2_. After electrophoresis, Phos-tag was soaked in transfer buffer containing 5 mM EDTA for 20 min twice, followed by another 10 min in buffer without EDTA to remove manganese ions from the gel before transferring to the polyvinylidene difluoride membrane.

### 2.5. Quantitative Reverse Transcription-Polymerase Chain Reaction

Total RNA was isolated using TRIzol (Vazyme, Nanjing, China) and applied to synthesize the first-strand cDNA at 25 °C for 10 min, 42 °C for 60 min and 70 °C for 15 min with random hexamers and M-MLV reverse transcriptase (Promega, Madison, WT, USA). The quantitative PCR was performed using the EzOmics SYBR qPCR Kit. Gene expression was calculated by 2^−ΔΔCt^ method, and the values were normalized to GAPDH or Rplp0 for human or mouse genes, respectively (primer sequences were shown in Table 1).

### 2.6. Immunoprecipitation and Ubiquitination Assays

HEK 293T cells were cotransfected with PXR and TRIM21. After 48 h, whole-cell lysates were prepared using RIPA supplemented with complete protease inhibitor PMSF. The lysates were immunoprecipitated using agarose beads coupled with rabbit anti-Flag or anti-TRIM21 antibodies or anti-IgG under the manufacturer’s protocol. The eluted proteins were run on 8% PAGE gel and transferred to a PVDF membrane. Membranes were blocked with 5% skim milk and incubated with rabbit anti-PXR or anti-TRIM21 overnight at 4 °C. After washing, the blots were incubated for 1 h with appropriate horseradish peroxidase-conjugated secondary antibody (Promega, Madison, WI, USA). For ubiquitination assays, cells were transfected with TRIM21 and HA tagged ubiquitin. Cell lysates were prepared as described previously and immunoprecipitated with rabbit-PXR antibody, and eluted proteins were probed with anti-HA antibody.

### 2.7. Immunofluorescence Analysis

To assay the expression of PXR, cells were seeded at 2 × 10^5^ cells per well on glass-bottom dishes and treated with indicated concentrations of dinaciclib or kenpaullone. At the end of incubation, cells were fixed for 30 min by 4% paraformaldehyde, permeabilized in 0.1% TritonX-100 for 30 min and blocked with 5% BSA for 1 h. Afterwards, cells were incubated with anti-PXR primary antibody at 4 °C overnight, followed by incubation with FITC-conjugated secondary antibody (Bioworld, Nanjing, China) in the dark for 1 h. Nuclei were stained with DAPI (Bioworld, Nanjing, China) for 10 min, and cells were examined with laser confocal microscopy.

### 2.8. Primary Hepatocyte Isolation

Primary hepatocytes were isolated from male C57BL/6 mice between 8 and 12 weeks of age. The surgery was performed under anesthesia to expose the hepatic portal vein. The liver was perfused through a hepatic portal vein cannula. The inferior vena cava was transected to allow perfusate outflow. The liver was sequentially perfused with the following solutions, first with 30 mL of HBSS with 0.65 mM EDTA (Solarbio, Beijing, China) and 0.075% NaHCO_3,_ followed by 10 mL of HBSS with 5 mM CaCl_2_, 0.075% NaHCO_3_ and 0.05% type IV collagenase (Biofroxx, Einhausen, Germany). All solutions were heated to 37 °C. Liver lobes were collected, taking care to avoid damage, and transferred to a Petri dish containing DMEM medium with 10% FBS and gently stirred to disperse the hepatocytes. The collected cells were washed twice with successive contrifugation at 50 g for 3 min. The debris and excess solution were aspirated, and the viable hepatocytes were resuspended in DMEM containing 10% FBS, counted, and subjected to cell culture.

### 2.9. Assay of Oxidative Activity of CYP3A4

HepG2 cells were placed into 96-well plates in DMEM with 10% FBS overnight and exposed to dinaciclib for 24 h. Then cells were treated using P450-Glo^TM^ Luminescent cytochrome P450 3A4 Assay System. Briefly, after cells were washed carefully, a mixture of DMEM and Luciferin-IPA was added to each well. After incubation for 60 min, 25 μL luciferin detection reagent was added to a 96-well opaque luminometer plate and incubated for 10 min in the dark. The luminescence signal was determined with a spectral scanning multimode reader.

### 2.10. Assay of Intracellular Accumulation of Rhodamine 123

The efflux activity of P-gp was determined by intracellular accumulation of a known P-gp substrate, rhodamine 123 (Rho 123). After the treatment, cells were placed with 5 μg·mL^−1^ Rho123 in DMEM (10% FBS) to incubate at 37 °C, 5% CO_2_ for 30 min in the dark. Cells were then washed twice with PBS and analyzed immediately by flow cytometry (BD FACSCalibur with Cellquest software, BD Biosciences, Franklin Lakes, NJ, USA) or observed under a fluorescent microscope.

### 2.11. Data and Statistical Analysis

The experimental results were expressed as the mean ± SEM. The differences between two groups were analyzed using a two-tailed Student’s t-test. Differences between more than two groups were analyzed by one-way ANOVA, followed by Dunnett’s post hoc test. The post hoc tests were run only if an overall statistically significant difference existed in group means, and there was no significant variance in homogeneity. *p* < 0.05 was considered statistically significant.

### 2.12. Materials

DMEM and FBS were from Gibco (Gibco, Grand Island, NY, USA). PD033291, kenpaullone (Ken), dinaciclib, okadaic acid, actinomycin D and rifampicin were purchased from Sigma (St. Louis, MO, USA), dissolved in DMSO to 100 mM and stored at −20 °C. The concentrations used in the study were 0.1~10 μM, freshly diluted with DMEM supplemented with 1% FBS (Gibco, Grand Island, NY, USA) to final concentrations. MTT was from SunShine Biotechnology (Nanjing, China). Phos-tag Acrylamide was purchased from Apexbio (Houston, TX, USA). Dual-Luciferase Reporter Assay System and P450-Glo^TM^ Luminescent cytochrome P450 3A4 Assay System were from Promega (Madison, WI, USA). Antibodies used in the present study were as follows: CES1 (Abcam, Cambridge, UK, ab68190), CES2 (Abcam, Cambridge, UK, ab184957), P-gp (Abcam, Cambridge, UK, ab170904), PXR (Abcam, Cambridge, UK, ab192579), DNAJC7 (Abcam, Cambridge, UK, ab179830), β-actin (Proteintech Technology, Wuhan, China, AF7018), Hsp90 (Proteintech Technology, Wuhan, China, 13171-1-AP), GAPDH (Proteintech Technology, Wuhan, China, 10494-1-AP), CYP3A4 (Proteintech Technology, Wuhan, China, 18227-1-AP), CDK2 (Proteintech Technology, Wuhan, China, 10122-1-AP), CDK5 (Proteintech Technology, Wuhan, China, 10430-1-AP), Ubiquitin (Proteintech Technology, Wuhan, China, 10201-2-AP), HA tag (Proteintech Technology, Wuhan, China, 51064-2-AP), Flag tag (Proteintech Technology, Wuhan, China, 66008-2-Ig), TRIM21 (ABclonal, Wuhan, China, A1957) and Phospho-MAPK/CDK Substrates (Cell Signaling Technology, Danvers, MA, USA, 2325S).

## 3. Results

### 3.1. The E3 Ligase TRIM21 Mediates the Ubiquitination and Degradation of PXR

**Figure 1:**
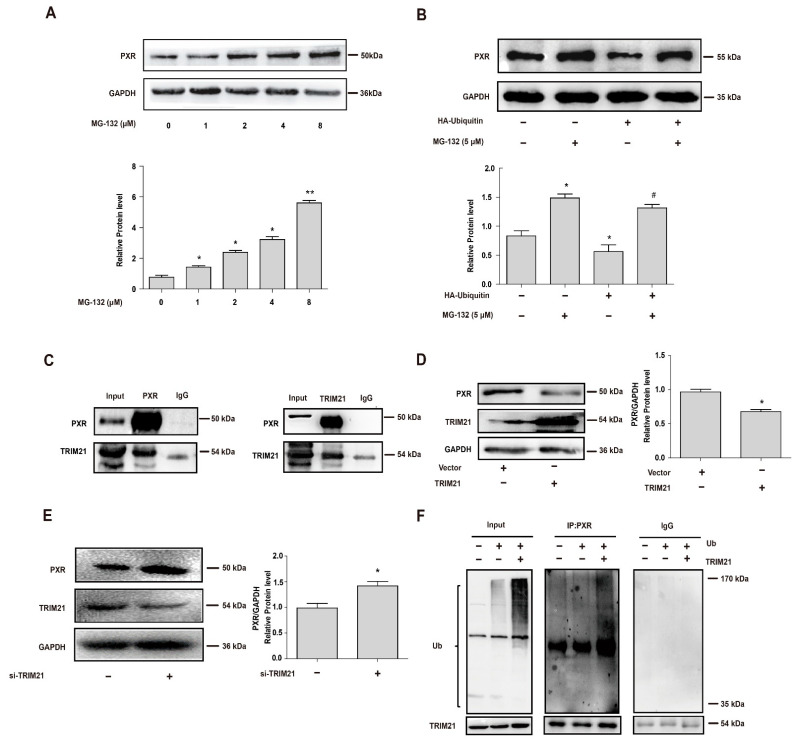
The E3 ligase TRIM21 binds to PXR and mediates its ubiquitination and degradation. (**A**) HepG2 cells were treated with MG-132 (0, 1, 2, 4 or 8 μM) for 6 h in the presence of CHX (10 μM), and PXR protein levels were determined by Western blot. (**B**) HEK 293T cells were transfected with HA-ubiquitin for 24 h and then treated with or without MG-132 5 μM for 24 h, and PXR protein levels were determined by Western blot. (**C**) Interaction of TRIM21 with PXR. HEK 293T cells were transfected with PXR and TRIM21 constructs as indicated. Cell extracts were immunoprecipitated using PXR antibody or IgG and probed with TRIM21 (left). Reciprocally, the extracts were immunoprecipitated with TRIM21 antibody or IgG and probed with PXR antibody (right). (**D**) Ectopic overexpression of TRIM21 in HepG2 cells decreased endogenous PXR levels. HepG2 cells were transfected with TRIM21 construct, and the protein level of PXR was analyzed by Western blot 48 h later. (**E**) HepG2 cells were transfected with siTRIM21 and siRNA-control for 48 h. Cell lysates were prepared and subjected to Western blot to determine the expression of PXR and TRIM21, respectively. (**F**) TRIM21 ubiquitinates PXR. HEK 293T cells were transfected with corresponding vector or HA-Ubiquitin with or without the co-transfection of TRIM21 as indicated. After 24 h, cells were treated with MG-132 (5 μM) for 24 h. Cell lysates were immunoprecipitated with PXR antibody (IP: PXR) or IgG, subjected to Western blot, and probed with anti-HA antibody to detect ubiquitination of PXR. The ubiquitinated ladder was more pronounced when the cells were transfected with ubiquitin and TRIM21 together, compared with those transfected with ubiquitin alone. Experiments described in this figure were repeated independently at least three times, and data are expressed as mean ± SEM (*n* = 3). * *p* < 0.05, ** *p* < 0.01 versus control; # *p* < 0.05 versus HA-ubiquitin overexpression group.

Stability of PXR was first analyzed using ubiquitin-proteasome pathway inhibitor MG-132 and ubiquitin-overexpressing plasmid. Protein level of PXR was determined after the cells were treated with different concentrations of MG-132 for 6 h in the presence of translation inhibitor cycloheximide (CHX, 10 μM). As shown, MG-132 effectively stabilized PXR protein against degradation in a concentration-dependent manner (Figure 1A). Then, HEK 293T cells were transfected with HA-ubiquitin construct or the corresponding vector for 24 h and then treated with MG-132 (5 μM) for another 24 h [31,32]. The protein level of PXR repeatedly increased with the treatment using MG-132. Additionally, ubiquitin overexpression significantly induced PXR degradation, which was potently abolished by MG-132 (Figure 1B).

It has been reported that TRIM21 is an E3 ubiquitin ligase and is known to target certain proteins for degradation in a proteasome-dependent manner [33]. Previous studies also found that TRIM21 was associated with PXR by searching for PXR-related proteins [18]. To further confirm that TRIM21 was a PXR-binding protein, we first ectopically co-transfected 293T cell with expression constructs for PXR and TRIM21. Cells were lysed and subjected to immunoprecipitation with nonspecific IgG or antibody to PXR or TRIM21 and subjected to Western blot analysis. As show in Figure 1C, TRIM21 was immunoprecipitated with PXR antibody, and conversely, PXR was also immunoprecipitated with TRIM21 antibody. Based on the indication that PXR was bound to TRIM21, we then investigated the effect of TRIM21 on the endogenous PXR protein levels in HepG2 cells. A statistically significant decrease in the endogenous level of PXR protein was observed by Western blot analysis followed by densitometric analysis when TRIM21 was ectopically overexpressed in HepG2 cells (Figure 1D). Similarly, we knocked TRIM21 down using siRNA in HepG2 cells, and the decreased expression of TRIM21 induced an increase in PXR protein levels (Figure 1E). These results suggested that TRIM21 decreased PXR protein levels by directly binding to it. Since TRIM21 is a well-known E3 ubiquitinating ligase, we used TRIM21-overexpressed cells to study TRIM21-mediated PXR ubiquitination [34,35]. We examined whether TRIM21 ubiquitinated PXR by co-transfection with expression plasmids for TRIM21 and HA-ubiquitin. Cell lysates were immunoprecipitated with anti-PXR antibody or IgG, and analyzed using Western blot with anti-HA antibody to detect HA-tagged ubiquitin and anti-TRIM21 antibody to confirm overexpression efficiency. It showed that ectopically overexpressed TRIM21 increased the ubiquitination of PXR with the overexpression of ubiquitin (Figure 1F). The data indicated that TRIM21 targeted PXR protein for proteasomal degradation by ubiquitination.

### 3.2. Inhibition of CDKs Increases the Protein Level of PXR through the Protein Ubiquitination and Proteasomal System

**Figure 2:**
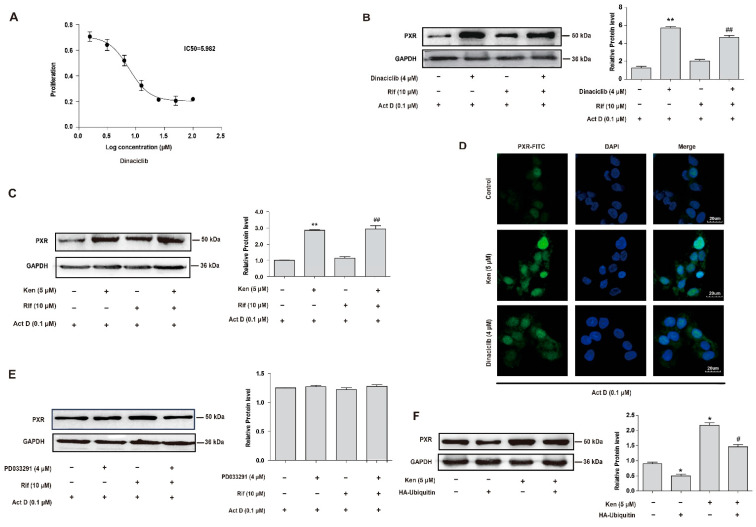
Inhibition of CDKs increases the protein level of PXR by suppressing its ubiquitination. (**A**) The cytotoxicity of dinaciclib in HepG2 cells. Hepatocytes were treated with indicated concentrations of dinaciclib for 24 h, and MTT assay was used to measure cell viability. (**B**–**E**) The effect of CDKs inhibitors on PXR protein stabilization in HepG2 cells. (**B**) Cells were treated with dinaciclib (4 μM) in the presence of actinomycin D (0.1 μM) with or without rifampicin (10 μM) for 24 h. (**C**) HepG2 cells were treated with kenpaullone (Ken, 5 μM) in the presence of actinomycin D (0.1 μM) with or without rifampicin (10 μM) for 24 h. (**D**) HepG2 cells were treated with kenpaullone (5 μM) or dinaciclib (4 μM) in the presence of actinomycin D (0.1 μM) for 24 h, and fluorescence intensity of PXR was detected by immunofluorescence analysis. (**E**) HepG2 cells were treated with PD033291 (4 μM) in the presence of actinomycin D (0.1 μM) with or without rifampicin (10 μM) for 24 h. PXR protein levels were investigated by Western blot. (**F**) HepG2 cells were transfected with HA-ubiquitin for 24 h and then treated with or without kenpaullone (5 μM) for 24 h. PXR protein levels were investigated by Western blot. Experiments described in this figure were repeated independently at least three times, and data are expressed as mean ± SEM (*n* = 3). * *p* < 0.05, ** *p* < 0.01 versus control; # *p* < 0.05 versus HA-ubiquitin overexpression group, ## *p* < 0.01 versus rifampicin-treated group.

PXR exists within the cells as a phosphoprotein, and its phosphorylation frequently connects to the suppression of PXR activity, with the involvement of several kinases, including PKA, CK2, CDK2 and CDK5 [36]. Therefore, the role of PXR phosphorylation-associated kinases in the stability of PXR was then investigated. Firstly, it was shown that the inhibition of PXR phosphorylation-associated kinases, including PD98059 (mitogen-activated protein kinase, MAPK inhibitor), LY294002 (Akt inhibitor), KN93 (CaMKII inhibitor), DRB (CK2 inhibitor), kenpaullone (ken, cyclin-dependent kinase 2, CDK2 inhibitor) and H89 (PKA inhibitor), significantly increased the protein level of PXR (Supplementary Figure S1). Next, kinase inhibitors were used in the presence of Actinomycin D (Act D, 0.1 μM) to exclude the influence of gene transcription. The results showed that CDK inhibitors exclusively increased the protein level of PXR with the suppression of gene transcription, indicating the involvement of protein degradation (Supplementary Figure S2). To further confirm the previous results, inhibitors of CDKs including PD033291 (specific inhibitor for CDK4 and 6), dinaciclib (specific inhibitor for CDK1, 2, 5 and 9, with relatively lower inhibition on CDK1 and 9) and kenpaullone (specific inhibitor for CDK1, 2 and 5) were used to evaluate different CDK subtypes. As kenpaullone and PD033291 had been widely used in a large number of studies with a functional concentration of 1~10 μM [37], we accordingly utilized an MTT cell viability assay to determine the effect of dinaciclib on HepG2 cells, as shown in Figure 2A. As shown in Figure 2B,C, the protein level of PXR was significantly increased by the treatment with kenpaullone (5 μM) or dinaciclib (4 μM) in the presence of Act D (0.1 μM), while no obvious trend was observed with the treatment using PD033291 (Figure 2E). It was noted that the increase in PXR protein levels induced by kenpaullone (5 μM) or dinaciclib (4 μM) was not affected by the treatment with rifampin, suggesting a ligand-independent pathway in CDK-mediated PXR destabilization (Figure 2B,C). Immunofluorescence analysis further verified the increased protein level of PXR by kenpaullone (5 μM) or dinaciclib (4 μM) in HepG2 cells. As shown in Figure 2D, PXR was increased in cytosol and nucleus after incubation with 5 μM kenpaullone or 4 μM dinaciclib for 24 h. The findings indicated that CDKs involved in the protein degradation of PXR were probably CDK1, 2 or 5. In this circumstance, we considered CDK2 and CDK5 as priority, since they induced a relatively higher effect compared with CDK1 by dinaciclib.

To investigate whether protein ubiquitination was involved in CDK inhibitor-suppressed PXR degradation, HepG2 cells were transfected with the HA-ubiquitin construct or the corresponding vector for 24 h and treated with kenpaullone (5 μM) or the same volume of vehicle (DMSO) for another 24 h. The efficiency of transfection was confirmed by analyzing the expression of PXR by Western blot (Figure 2F). The protein level of PXR was decreased in the cells transfected with HA-ubiquitin, but the treatment with kenpaullone (5 μM) significantly abolished the reduced PXR protein caused by HA-ubiquitin (Figure 2F). The data confirmed that CDK was essential in the promoted degradation of PXR through the ubiquitin–proteasome pathway.

### 3.3. The Attenuated Interaction of PXR with DNAJC7 and Hsp90 Is Engaged in PXR Degradation by CDKs

**Figure 3:**
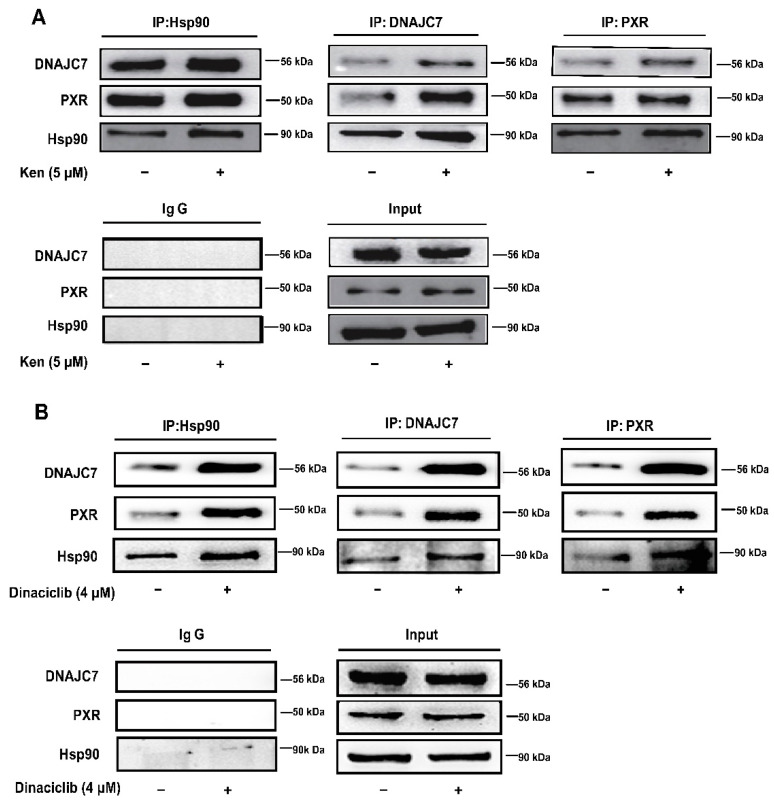
The CDK inhibitors increase the interaction between PXR, DNAJC7 and Hsp90. HepG2 cells were treated with kenpaullone (5 μM) (**A**) or dinaciclib (4 μM) (**B**) for 24 h, and the interactions of PXR with DNAJC7 and Hsp90 were investigated by co-immunoprecipitation. Cell lysates were immunoprecipitated with anti-PXR antibody, anti-Hsp90 antibody or anti-DNAJC7 antibody, followed by immunoblotting with anti-PXR antibody, anti-Hsp90 antibody or anti-DNAJC7 antibody, respectively. Experiments described in this figure were repeated independently at least three times.

The cytoplasmic CAR retention protein (DNAJC7) had been shown to maintain the cytoplasmic localization of CAR and PXR by forming a complex with the NRs and Hsp90 [16,38]. Therefore, we investigated whether the formation of a complex with PXR, Hsp90 and DNAJC7 in the cytoplasm plays a role in CDK-promoted PXR degradation. Immunoprecipitation with the specific anti-PXR, anti-Hsp90 and anti-DNAJC7 antibody revealed that the CDK inhibitors significantly promoted the association of PXR with Hsp90 and DNAJC7. The formation of the PXR-Hsp90-DNAJC7 complex was increased after the treatments with kenpaullone (5 μM) and dinaciclib (4 μM) (Figure 3A,B) respectively, which suggested that CDKs dissociated PXR from the complex with DNAJC7 and Hsp90, and subsequently promoted PXR degradation.

### 3.4. CDKs, and CDK2 Specifically, Decrease PXR Protein Stabilization and Suppress the Expression of PXR-Target Genes

**Figure 4:**
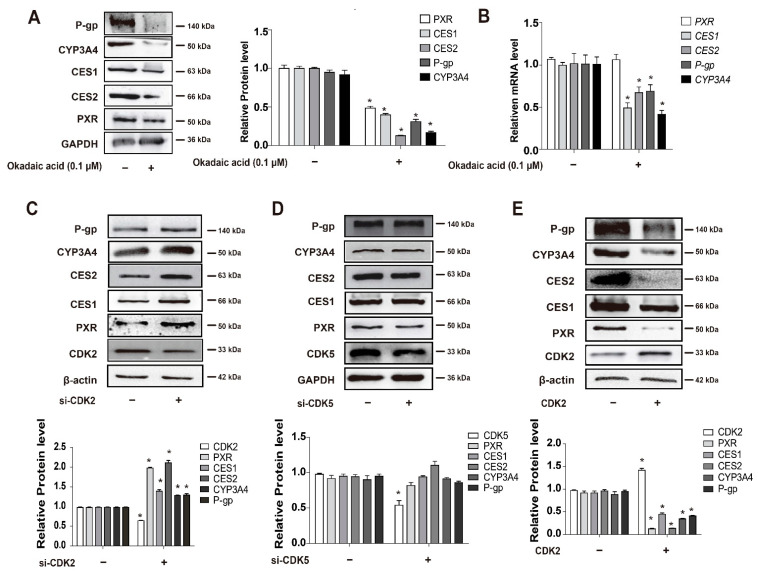
CDK2-mediated phosphorylation destabilizes PXR protein and suppresses PXR-targeted genes expression. (**A**,**B**) HepG2 cells were treated with okadaic acid (0.1 μM) for 24 h, and the gene expression was analyzed at protein (**A**) or mRNA levels (**B**). Cell lysates were prepared and subjected to Western blot to determine the expression of PXR, CES1, CES2, CYP3A4 and P-gp, respectively. mRNA levels were detected by qRT-PCR. (**C,D**) HepG2 cells were transfected with si-CDK2 or si-CDK5 and siRNA-control for 48 h. Cell lysates were prepared and subjected to Western blot to determine the expression of PXR, CES1, CES2, CYP3A4 and P-gp, respectively. (**E**) The effect of CDK2 overexpression on the suppression of CES1, CES2, PXR, CYP3A4 and P-gp. Experiments described in this figure were repeated independently at least three times, and data are expressed as mean ± SEM (*n* = 3). * *p* < 0.05 indicates significant difference compared with control group.

Subsequently, the experiments were designed to investigate whether the phosphorylation of PXR was involved in the promoted PXR degradation by CDKs. After the treatment with the protein phosphatase (PP) inhibitor okadaic acid at 100 nM, PXR and its target genes’ protein expression were significantly downregulated (Figure 4A). The mRNA expression of PXR-target genes was significantly downregulated by okadaic acid (100 nM), while the mRNA level of PXR was not obviously altered (Figure 4B). This strongly indicated that it was PXR degradation that mediated the downregulation of PXR-target genes with the increased phosphorylation status of PXR. Since it had been proposed that CDK2 and CDK5 catalyzed the phosphorylation of PXR and affected its transcriptional activity [24,39], we further focused on the role of CDK2 and CDK5 in the PXR degradation. We depleted CDK2 or CDK5 respectively to examine which of the CDKs affected PXR degradation and expression of its target genes. CDK2 depletion induced the increased protein level of PXR and led to elevated expression of PXR-target genes (Figure 4C). Conversely, overexpression of CDK2 decreased the protein level of PXR and the expression of PXR-target genes (Figure 4E), whereas no significant change in the protein level of PXR and expression of its target genes was observed when CDK5 was depleted (Figure 4D). The results provided evidence that CDK2-mediated phosphorylation promoted PXR degradation and subsequently decreased PXR-target gene expression.

### 3.5. CDKs Inhibitor Dinaciclib Promotes the Expression and Activity of PXR Target Genes in Primary Hepatocytes and HepG2 Cells

**Figure 5:**
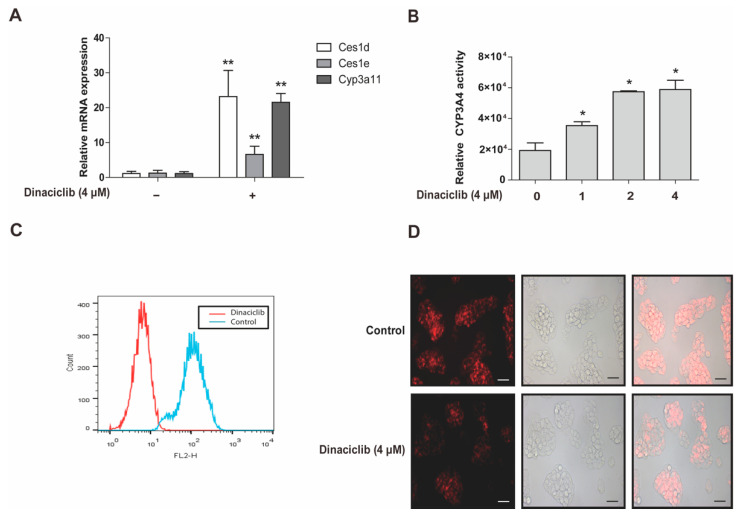
CDK2 inhibitor dinaciclib increases the expression and activity of PXR target genes. (**A**) Primary mouse hepatocytes were treated with dinaciclib (4 μM) for 24 h, and the gene expression was analyzed at mRNA levels. (**B**) Dinaciclib promoted the oxidative activity of CYP3A4. HepG2 cells were treated with dinaciclib (0, 1, 2 or 4 μM) for 24 h, and cell lysates were prepared and assayed for CYP3A4 activity. (**C**,**D**) Dinaciclib promoted the efflux activity of P-gp. HepG2 cells were treated with or without dinaciclib (4 μM) for 24 h, then incubated with DMEM (10% FBS) supplemented with 5 μg·mL^−1^ Rho 123 for 30 min. The efflux activity of P-gp was analyzed immediately by flow cytometry (**C**) or observed under a fluorescence microscope (**D**). Scale bar: 50 μm. Experiments described in this figure were repeated independently at least three times, and data are expressed as mean ± SEM (*n* = 3). * *p* < 0.05 and ** *p* < 0.01 indicates significant difference compared with control group.

Carboxylesterases, CYP3A4 and P-gp are classical target genes of PXR [2]. In order to further explore the potential roles of CDKs in regulating DMETs and contribute to the possibility of predicting DDIs in clinical therapy, dinaciclib was exposed to primary mouse hepatocytes. The gene expression of Cyp3a11, Ces1d and Ces1e, which resemble human CYP3A4, CES1 and CES2 in terms of enzymatic function and transcriptional regulation, was analyzed in primary mouse hepatocytes [4,40,41]. The mRNA expression of Cyp3a11, Ces1d, Ces1e genes was significantly upregulated by dinaciclib (4 μM) (Figure 5A). The activity of CYP3A4 and P-gp was then measured in HepG2 cells after exposure to dinaciclib. The oxidative activity of CYP3A4 was markedly increased by dinaciclib in a concentration-dependent manner (Figure 5B). Moreover, the efflux activity of P-gp was analyzed by Rho 123 intracellular accumulation assay with flow cytometry as well as fluorescence microscope. In the cells pretreated with dinaciclib (4 μM), intracellular Rho 123 accumulation was markedly decreased, compared with the cells treated with vehicle (Control). It was shown that the efflux activity of P-gp was significantly increased (Figure 5C,D). The data supported that the expression and activity of PXR-targeted genes in hepatocytes were activated by dinaciclib. This implies a possible effect on the range of DDIs induced by CDK inhibitors when used in clinical settings.

### 3.6. PXR Phosphorylation at S350 by CDK2 Triggers PXR Degradation via the Ubiquitin-Proteasome Pathway

**Figure 6:**
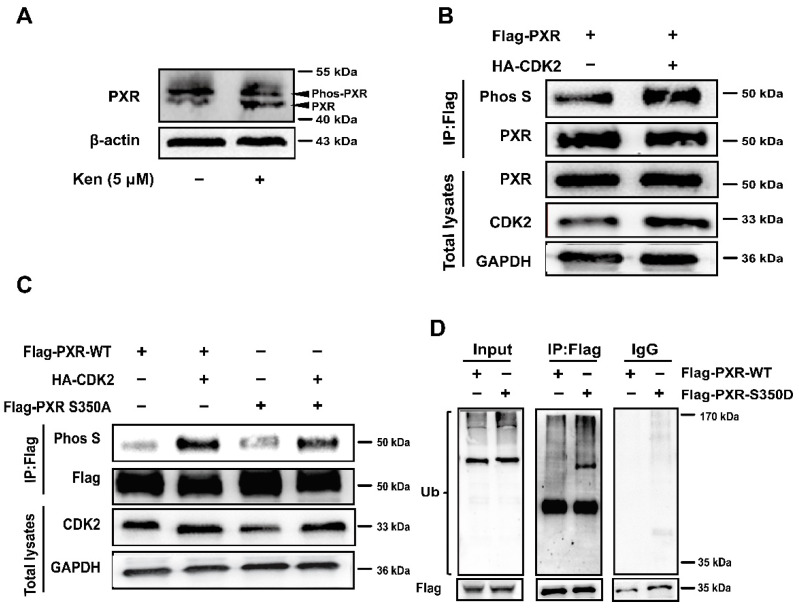
PXR phosphorylation at S350 by CDK2 triggers PXR degradation via ubiquitin-proteasome pathway. (**A**) HepG2 cells were treated with kenpaullone (5 μM) for 24 h. Cytoplasmic fractions (30 μg each) were subjected to Phos-tag SDS-PAGE, and the levels of phosphorylated PXR and non-phosphorylated PXR were analyzed. (**B**) HEK 293T cells were transfected with full-length Flag-PXR with or without the co-transfection of HA-CDK2. Cell lysates were immunoprecipitated with anti-Flag and immunoblotted with anti-phos serine. (**C**) HEK 293T cells were transfected with Flag-PXR or Flag-PXR-S350A (Ser350A) with or without the co-transfection of HA-CDK2. Cell lysates were immunoprecipitated with anti-Flag antibody, then immunoblotted with anti-phos serine antibody. (**D**) HEK 293T cells were co-transfected with HA-ubiquitin and Flag-PXR (WT) or Flag-PXR-S350D (Ser350D). After 24 h, cells were treated with 5 μM MG-132 for 24 h. Ubiquitination of WT-PXR or Ser350D-PXR was measured by IP-immunoblotting. Experiments described in this figure were repeated independently at least three times.

It had been shown that Ser350 of PXR, a putative phosphorylation site, is crucial for PXR heterodimerization with RXRα and the resulting transcriptional activation of PXR-target genes [39]. Since the binding of Phos-tag increased the molecular weight of phosphorylated PXR, it could be separated from the dephosphorylated one on the gels. The treatment with kenpaullone (5 μM) increased the dephosphorylated levels of PXR on the Phos-tag gels, while decreasing the phosphorylated PXR (Figure 6A). Furthermore, 293T cells were co-transfected with full-length Flag-PXR with or without HA-CDK2, and evaluated for the phosphorylation of PXR using anti-phospho-serine antibody. It was demonstrated that PXR phosphorylation increased in the presence of HA-CDK2 (Figure 6B). To determine the effect of phosphorylation on PXR ubiquitination, we designed a mutant phosphomimetic form of PXR by mutating Ser350 to aspartate (S350D) and a phosphodeficient mutation from Ser350 to alanine (S350A). The Ser350A mutant PXR had slightly lower phosphorylation levels in vitro compared with WT PXR when overexpressing CDK2 (Figure 6C). Then, WT or S350D PXR was transfected into the cells and incubated with MG-132 (5 μM) to block proteasome degradation. The PXR-S350D mutant showed higher ubiquitination when compared with the PXR-WT (Figure 6D). Thus, phosphorylation at Ser350 of PXR by CDK2 triggered the ubiquitination of PXR and its subsequent degradation via the ubiquitin-proteasome pathway.

## 4. Discussion

Nuclear receptors respond to extracellular and intracellular substances and mediate transcriptional regulation of target gene expression to participate in many physiological processes of the organism, such as drug metabolism [42]. Therefore, studying the post-translational modification of PXR is essential to the development of new drugs and the understanding of drug interactions. The phosphorylation of PXR mediated by various protein kinases, including PKA, CaMKII and p70S6K, leads to the promoted interaction with corepressors and inhibits the transcriptional activity of PXR [15,43,44]. In the present study, we identified a novel negative-regulating mechanism of PXR involving CDK2-mediated phosphorylation-dependent degradation. This conclusion is based on the following findings that the protein level of PXR is significantly increased by treatment with kenpaullone (5 μM) or dinaciclib (4 μM) in the presence of Act D (0.1 μM) (Figure 2B,C), and MG132 effectively stabilizes PXR protein against ubiquitin-dependent degradation (Figure 1B). A series of following experiments suggest that CDK2 directly phosphorylates PXR at Serine 350 in vitro (Figure 6C,D), subsequently destabilizes PXR and decreases its transcriptional activity (Figure 4 and Figure 5). This is supported by a previous study suggesting that S350 residue has the ability to affect PXR conformation and intramolecular interaction, resulting in interference with PXR-RXR dimerization [45]. The new findings have vital significance for an in-depth understanding of the negative regulation of PXR.

As many proteolytic pathways exist in regulating the stability of PXR, the ubiquitin-proteasomal pathway is one of the most frequently reported [3]. The overexpression of HA-ubiquitin eliminates the increase in PXR protein levels induced by the CDK inhibitor Ken (Figure 2F), indicating that ubiquitination is involved in the suppressed PXR degradation by CDK inhibitors. The ubiquitination inhibitors (such as MLN7243) in combination would be a good supplement to confirm the role of ubiquitination in the post-translational regulation of PXR [46]. RBCK1 (Ring-B-box-coiled-coil protein interacting with protein kinase C-1) is an E3 ligase that is previously reported to ubiquitinate PXR and induce its degradation through the ubiquitin-proteasome pathway [3]. We found that another E3 ligase, TRIM21, directly binds to PXR and mediates its degradation (Figure 1C–F). Our study confirmed the ubiquitination effect of TRIM21 on PXR stabilization using overexpressing and knockdown cells.

It was demonstrated that protein phosphatase (PP) inhibitor okadaic acid (100 nM) represses the transcriptional expression of all PXR-targeted genes by decreasing the protein level of PXR (Figure 4A,B). This indicates a phosphorylation-related destabilization of PXR, since the mRNA level of PXR is not suppressed by okadaic acid (Figure 4B). These results are consistent with a previous report that pointed out that okadaic acid (100 nM) suppresses CYP3A4 mRNA in HepG2 cells without providing a mechanistic explanation [44]. Decreased proliferation of hepatoma/hepatocarcinoma cells HepG2 or HUH7 by CDKs or MAPK kinase inhibitors is frequently associated with the increase in PXR-mediated regulation [13,14], which is consistent with the current findings that CDK inhibitors suppress PXR degradation through the regulation of phosphorylation. Our data go one step further by addressing the underlying mechanisms of PXR inhibitory machinery. In addition, we found that the inhibition of CDKs promotes the interaction of PXR with DNAJC7 and Hsp90 (Figure 3A,B), which helps avoid the binding of ubiquitin and subsequent degradation [47]. Even though HepG2 cells are frequently used to study the functions and mechanisms of PXR [20,48,49], it is essential to describe the mechanisms of PXR regulation using primary hepatocytes or non-proliferative and differentiated HepaRG cells. Primary mouse hepatocytes are cell models very commonly used for studying the transcriptional regulation of drug-metabolizing enzymes [4,41,50]. Since CDKs are more active as a cell cycle modulator in hepatoma cells compared with normal non-proliferative cells, primary mouse hepatocytes were also applied to further elucidate the effect of CDK inhibitor dinaciclib on PXR-targeted DMET gene expression in the present study (Figure 5A).

Dysregulated activity of CDKs, mostly due to a lack of endogenous CDK inhibitors or increased expression of cellular cyclins, has been shown to be a critical factor resulting in the loss of cell cycle control in human cancers [51,52,53]. Although they were discovered years ago, CDK inhibitors have recently been considered for important antineoplastic therapies. First-generation CDK inhibitors, such as flavopiridol and roscovitine, have a narrow therapeutic window and low selectivity [23]. New CDK inhibitors are in development, and some of these inhibitors have already been tested in clinical trials for the treatment of cancer. Dinaciclib is designed to maximize the therapeutic index and minimize the toxicity associated with CDK inhibitors [54]. Some researches indicate that chemotherapy using CDK4 inhibitors in combination with doxorubicin increases pro-apoptotic protein expression [55]. Additional study shows that palbociclib results in increased breast cancer cell cycle arrest together with anastrozole [56]. A novel CDK2/9 inhibitor, CYC065, was applied with eribulin to suppress the growth of triple negative breast cancer cells [57]. Considering the wide application of CDK inhibitors in cancer therapy, rational approaches to medical treatment will provide better benefits for patients.

Dinaciclib has potential clinical applications in the treatment of various cancer types, including chronic lymphoblastic leukemia [58], lung cancer [59], breast cancer [60] and neuroblastoma [61]. In a phase I study, 60% of the patients experienced severe adverse events [62], and in a subsequent phase II study, the fraction increased to 74% [63]. Therefore, understanding the molecular mechanisms of CDK inhibitors will facilitate rational applications and reduce adverse drug reactions. As a promising CDK inhibitor, the potential DDIs of dinaciclib are of more importance to be known than those of another CDK inhibitor, Ken, which we used in the present study. Our data provide evidence to possibly predict the DDIs of dinaciclib by exploring its potential role in regulating DMEs and drug transporters, including CYP3A4 and P-gp (Figure 5). The present finding that the expression and oxidative activity of CYP3A4 is markedly increased by dinaciclib will be important for the formulation of optimal dosage regimens in clinical cancer therapy (Figure 5B), since CYP3A4 metabolizes approximately 60% of therapeutic drugs and its inhibition frequently causes unfavorable DDIs and toxicity [64]. In addition, dinaciclib is primarily metabolized by CYP3A4/5 [65], and the therapeutic effects of dinaciclib may be affected by coadministration with other drugs that inhibit or induce the CYP3A4 pathway [65]. It is thus necessary to evaluate the influence on the metabolism of other drugs to avoid DDI-induced adverse reactions, in the development of new dinaciclib analogues.

ATP-binding cassette (ABC) efflux transporters protectively limit the entry of xenobiotics into biological membranes, thus possibly controlling drug penetration into particularly sensitive organs [66]. Inhibitors and substrates of P-gp, a member of the ABC transporter family, participate in pharmacokinetic DDIs that affect both therapeutic efficacy and the severity of adverse effects. Dinaciclib is a substrate of P-gp. The efflux of dinaciclib by P-gp reduces its anti-proliferative effects, which is probably the causative mechanism of cellular resistance to dinaciclib [67]. It has been shown that the expression and efflux activity of P-gp is significantly increased after the treatment with dinaciclib for 24 h (Figure 5C,D). Research indicates that ABC transporters diminish the accumulation of dinaciclib in cancer cells, leading to the development of drug resistance and subsequent failure of anticancer therapy [67]. Thus, the DDIs of dinaciclib with P-gp substrate chemotherapeutics might be an important cause of the decreased efficacy of substrate chemotherapeutics. We found that dinaciclib regulates CYP3A4 and P-gp through PXR, which provides new ideas for studying the DDIs of dinaciclib (Figure 5B–D). Our current findings about the induction of CYP3A4 expression by dinaciclib were also confirmed in primary hepatocytes in a cell-autonomous way (Figure 5A). Whether the induction of CYP3A4/P-gp in hepatocytes will lead to DDIs at the overall pharmacokinetic level still warrants further study [68].

It has been shown that some protein kinases regulate the protein level of PXR. In the present study, kinase inhibitors were used in the presence of Act D (0.1 μM) to exclude the influence of gene transcription. The results showed that no obvious trend was observed with treatments using PD98059, LY294002 and KN93 (Supplementary Figure S2), indicating that MAPK, Akt and CaMKII probably decrease the protein level of PXR by suppressing PXR transcriptional expression. They also show that H89 and DRB exclusively decrease the protein level of PXR (Supplementary Figure S2). A previous study echoes that CK2 promotes Hsp90β phosphorylation, which forms a complex with PXR to protect it from degradation and improve its stability [47]. The mechanism of PKA regulating the expression of PXR is still not clear to date. The results demonstrate that mechanisms of different kinases regulating PXR are still not well understood and need further study, which will provide new approaches for a better understanding of DDIs in clinical trials.

In summary, our research was divided into three stages. First, the phosphorylated PXR was identified by the E3 ubiquitin ligase, TRIM21, which promotes protein degradation of PXR through the ubiquitin-proteasome pathway. Second, we verified that CDK2 promotes the dissociation of DNAJC7 and Hsp90 by phosphorylation of PXR at S350. Third, CDK inhibitor dinaciclib increases the expression and activity of CYP3A4 and P-gp, which are targets of PXR (shown in graphic abstract). The findings have important implications for elucidating a novel molecular mechanism of CDK2-mediated suppression of PXR and providing some theoretical explanation with respect to the DDIs in cancer therapy, and ultimately indicate that CDK2-inhibitors might induce a wide range of DDIs in a PXR-related way.

## Figures and Tables

**Table 1:** Nucleotide Sequences of Gene-Specific Primers Used for Quantitative Real-Time PCR, Related to the Experimental Procedures.

Species	Gene	Sequence of Forward and Reverse Primers
Homo sapiens	*GAPDH*	Forward 5′-AAGGTCGGAGTCACCGGATT-3′
		Reverse 5′-CTGGAAGATGGTGAGGGATT-3′
Homo sapiens	*CES1*	Forward 5′-CCAGAGAGAGTCAACCCCTTCT-3′
		Reverse 5′-TCCTGCTTGTTAATTCCGACC-3′
Homo sapiens	*CES2*	Forward 5′-ACCGCAGTGGAGTCAGAGTTTC-3′
		Reverse 5′-ATGCTGAGGTACAGGCAGTCCT-3′;
Homo sapiens	*PXR*	Forward 5′-GGCAATCCCAGGTTCTCTTT-3′
		Reverse 5′-ATGCTTTATGGCAGGTGAGG-3′
Homo sapiens	*CYP3A4*	Forward 5′-TTCAGCAAGAAGAACAAGGACAA-3′
		Reverse 5′-GGTTGAAGAAGTCCTCCTAAGC-3′
Homo sapiens	*MDR1*	Forward 5′-GAGGCCAACATACATGCCTTC-3′
		Reverse 5′-GTCTAACAAGGGCACGAGCTAT-3′
Mus musculus	Ces1d	Forward 5′-GAGACCCAAGGCAGTAATAGGA-3′
		Reverse 5′-GAGTTGAGGCACCAATCTTCA-3′
Mus musculus	Ces1e	Forward 5′-CCAGTGACAGGGCAAATAGTC-3′
		Reverse 5′-TCATGCGTAGACAGGACCAGT-3′
Mus musculus	Cyp3a11	Forward 5′-ACAGCACTGGTCAGAGCCTGAA-3′
		Reverse 5′-GAGAGCAAACCTCATGCCAAGG-3′
Mus musculus	Rplp0	Forward 5′-GAAACTGCTGCCTCACATCCG-3′
		Forward 5′-GCTGGCACAGTGACCTCACACG-3′

## Data Availability

Not applicable.

## Supplementary Materials

The following supporting information can be downloaded at: https://www.mdpi.com/article/10.3390/cells11020264/s1, Figure S1: The effect of kinase inhibitors on the PXR protein level in HepG2 cells; Figure S2: The effect of kinase inhibitors on the PXR protein level in HepG2 cells in the presence of transcription inhibitor actinomycin; original Western blot data.

## Abbreviations

PXR	pregnane X receptor
CYP3A4	human cytochrome P450 family 3 subfamily A member 4
CES1	human carboxylesterase 1
CES2	human carboxylesterase 2
P-gp	P-glycoprotein
DMET	drug-metabolizing enzymes and transporters
DDIs	drug-drug interactions
Cyp3a11	mouse cytochrome P450 family 3 subfamily A member 11
Ces1d	mouse carboxylesterase 1
Ces1e	mouse carboxylesterase 2
