# Supplementary material for: Phosphorylation-Induced Ubiquitination and Degradation of PXR through CDK2-TRIM21 Axis
Source: Cells. 2022 Jan 13;11(2):264. doi: 10.3390/cells11020264 (PMC8773821; doi:10.3390/cells11020264)
Supplement: Supplementary file 1 [file cells-11-00264-s001.zip › cells-1358804-Supplementary Materials/cells-1358804-Supplementary Figures.pdf]

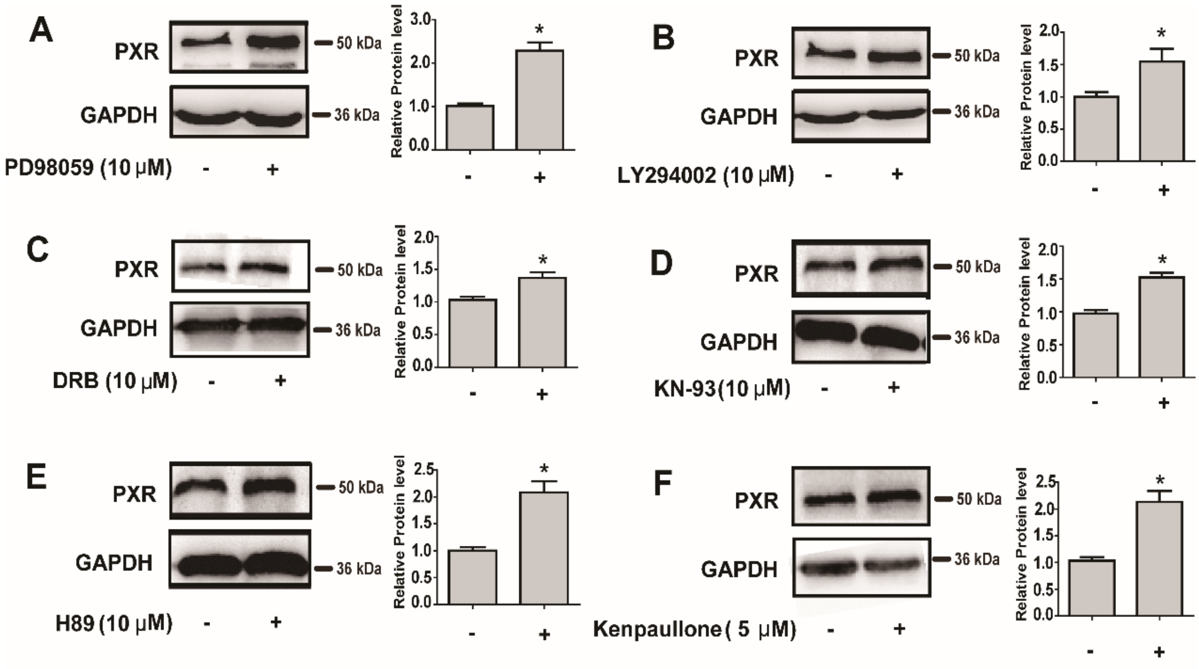

Figure S1: The effect of kinase inhibitors on the PXR protein level in HepG2 cells. Cells were treated with PD98059 (10 μM) (**A**), LY294002 (10 μM) (**B**), DRB (10 μM) (**C**), KN-93 (10 μM) (**D**), H89 (10 μM) (**E**) or Kenpaullone (5 μM) (**F**) for 24 h. PXR protein levels were investigated by Western blot. Experiments described in this figure were repeated independently at least three times, and data are expressed as mean ± SEM (n = 3). *P < 0.05 indicates significantly difference compared with control group.

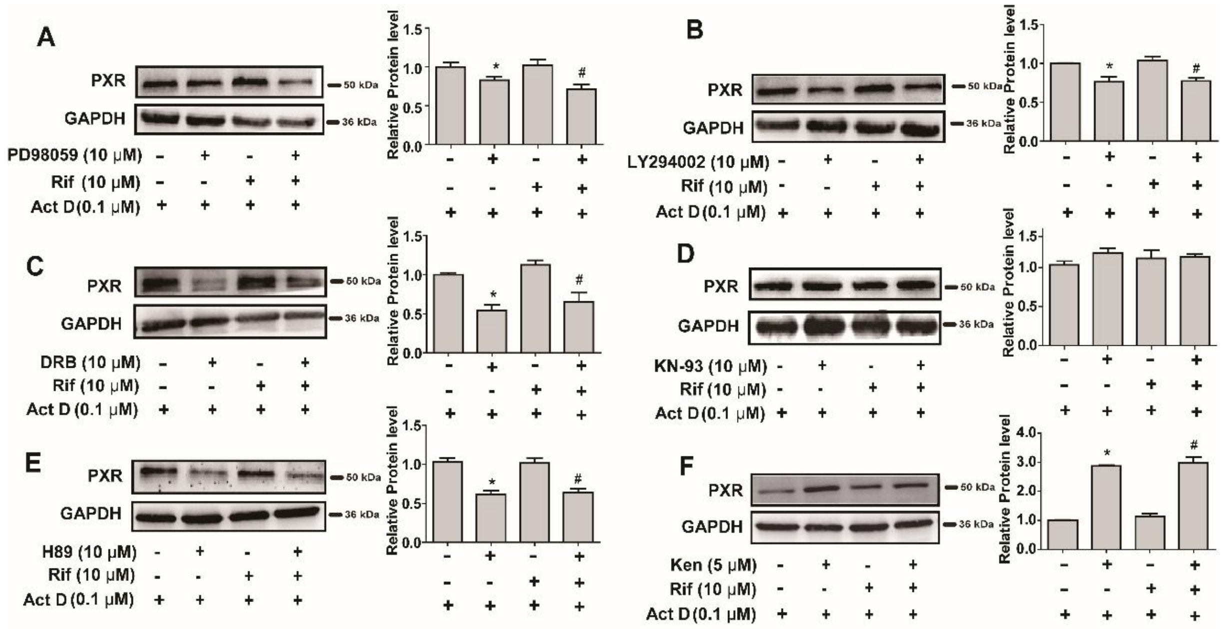

Figure S2: The effect of kinase inhibitors on the PXR protein level in HepG2 cells in the presence of transcription inhibitor actinomycin D. HepG2 cells were treated with PD98059 (10 μM) (**A**), LY294002 (10 μM) (**B**), DRB (10 μM) (**C**), KN-93 (10 μM) (**D**), H89 (10 μM) (**E**) or Kenpaullone (5 μM) (**F**) in the presence of actinomycin D (0.1 μM) with or without rifampicin (10 μM) for 24 h. PXR protein levels were investigated by Western blot. Experiments described in this figure were repeated independently at least three times, and data are expressed as mean ± SEM (n = 3). *P < 0.05 versus control group, #P < 0.05 versus rifampicin-treated group.
