# Supplementary material for: Phosphorylation-Induced Ubiquitination and Degradation of PXR through CDK2-TRIM21 Axis
Source: Cells. 2022 Jan 13;11(2):264. doi: 10.3390/cells11020264 (PMC8773821; doi:10.3390/cells11020264)
Supplement: Supplementary file 1 [file cells-11-00264-s001.zip › cells-1358804-Supplementary Materials/cells-1358804-Supplementary Western Blots Data.pdf]

Figure 1

A

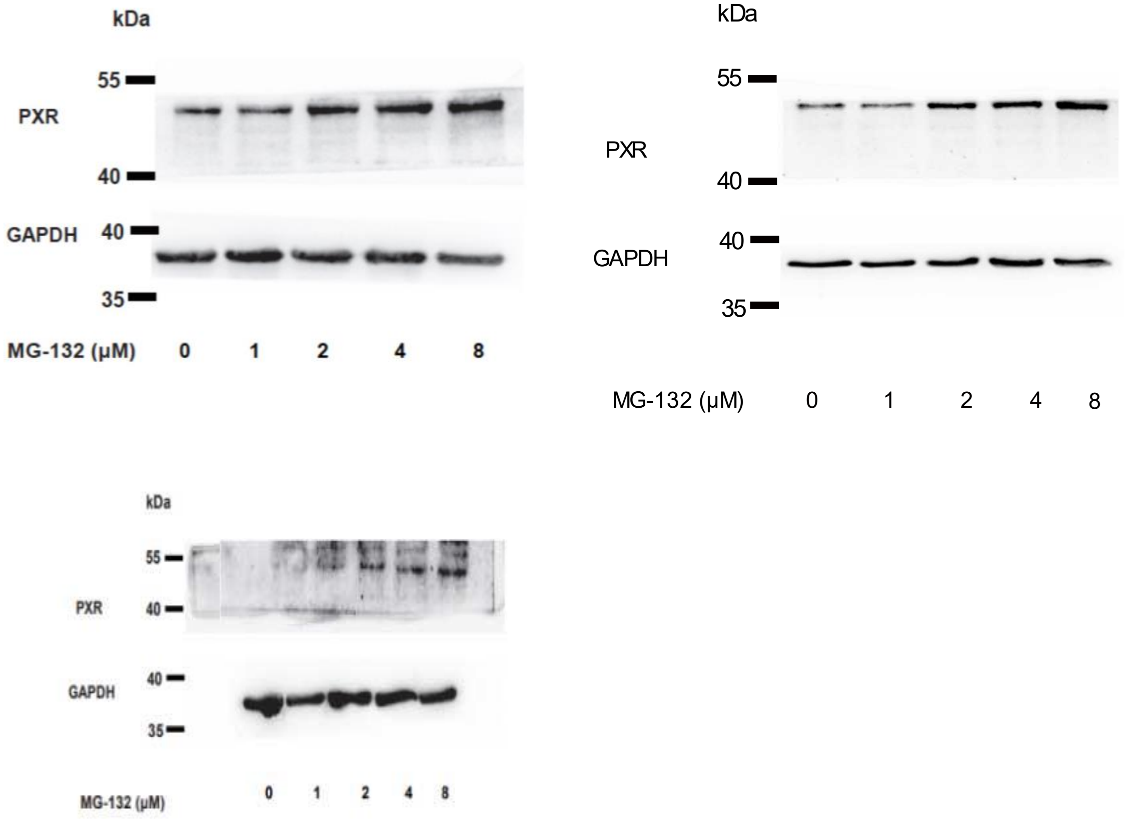

kDa
55
PXR
40
GAPDH
40
35
MG-132 (μM)
0
1
2
4
8
kDa
55
PXR
40
40
GAPDH
35
MG-132 (μM)
0
1
2
4
8
kDa
55
PXR
40
40
GAPDH
35
MG-132 (μM)
0
1
2
4
8

B

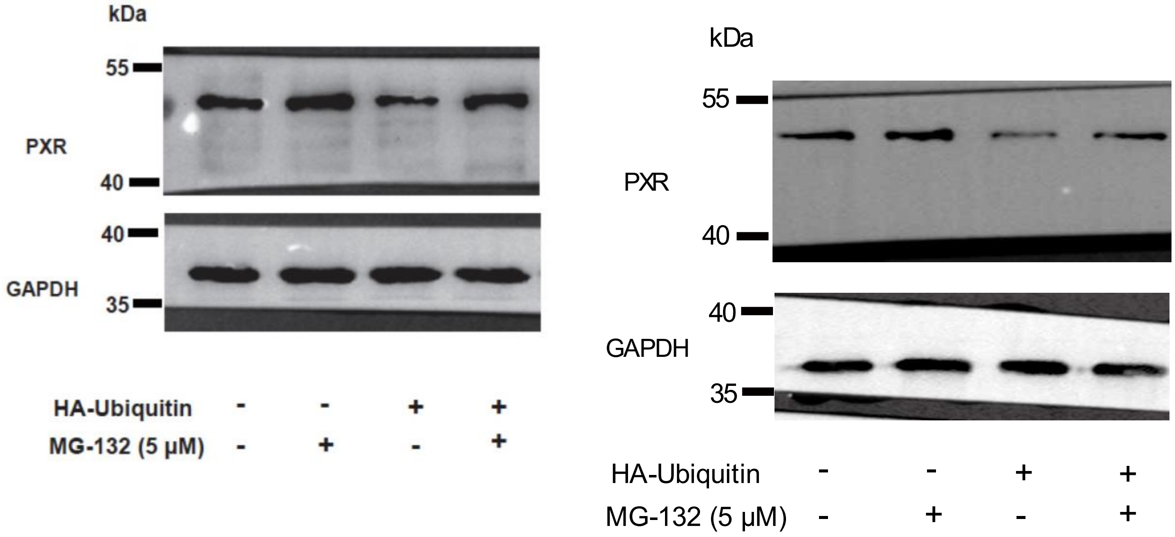

kDa
55
PXR
40
40
GAPDH
35
HA-Ubiquitin - - + +
MG-132 (5 μM) - + - +
kDa
55
PXR
40
40
GAPDH
35
HA-Ubiquitin - - + +
MG-132 (5 μM) - + - +

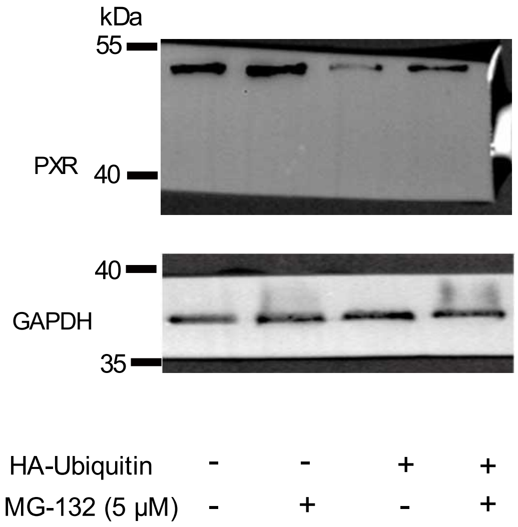
kDa
55
40
PXR
40
35
GAPDH
HA-Ubiquitin - - + +
MG-132 (5 µM) - + - +

C

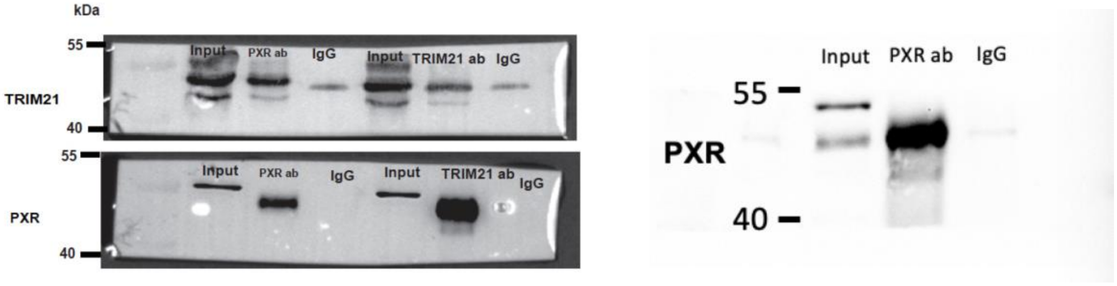
kDa
55
40
TRIM21
Input
PXR ab
IgG
Input
TRIM21 ab
IgG
PXR
55
40
Input
PXR ab
IgG
55
40
PXR

D

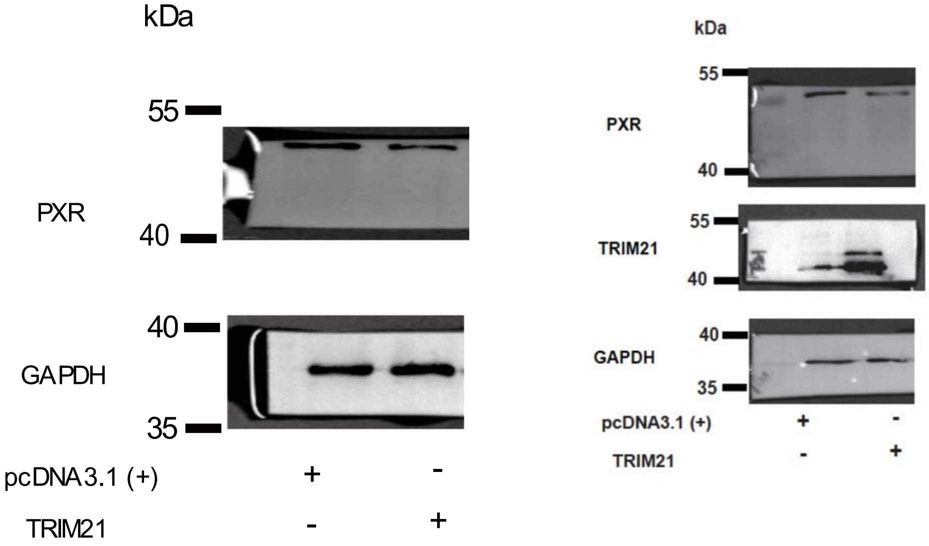
kDa
55
40
PXR
40
35
GAPDH
pcDNA3.1 (+) + -
TRIM21 - +
kDa
55
40
PXR
55
40
TRIM21
40
35
GAPDH
pcDNA3.1 (+) + -
TRIM21 - +

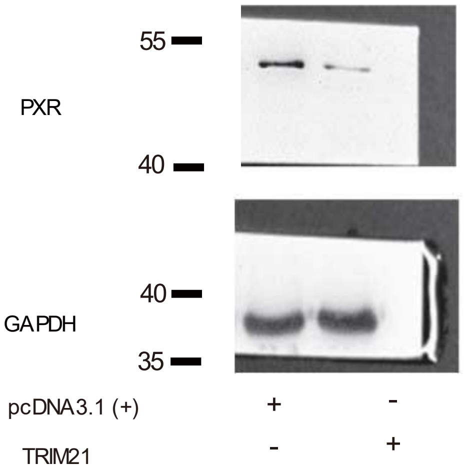
55
PXR
40
40
GAPDH
35
pcDNA3.1 (+)
+
-
TRIM21
-
+

E

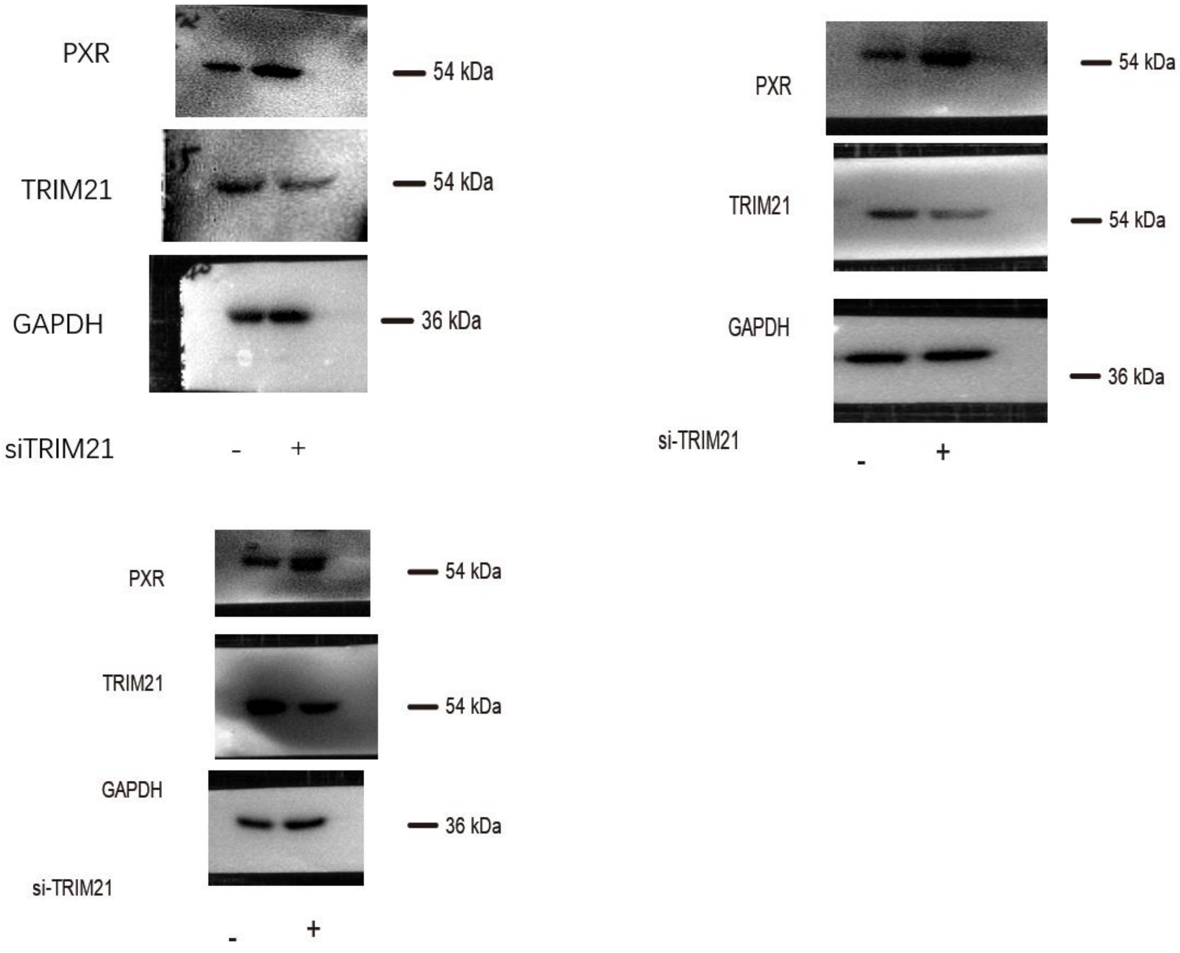
PXR
54 kDa
TRIM21
54 kDa
GAPDH
36 kDa
siTRIM21
-
+
PXR
54 kDa
TRIM21
54 kDa
GAPDH
36 kDa
si-TRIM21
-
+
PXR
54 kDa
TRIM21
54 kDa
GAPDH
36 kDa
si-TRIM21
-
+

F

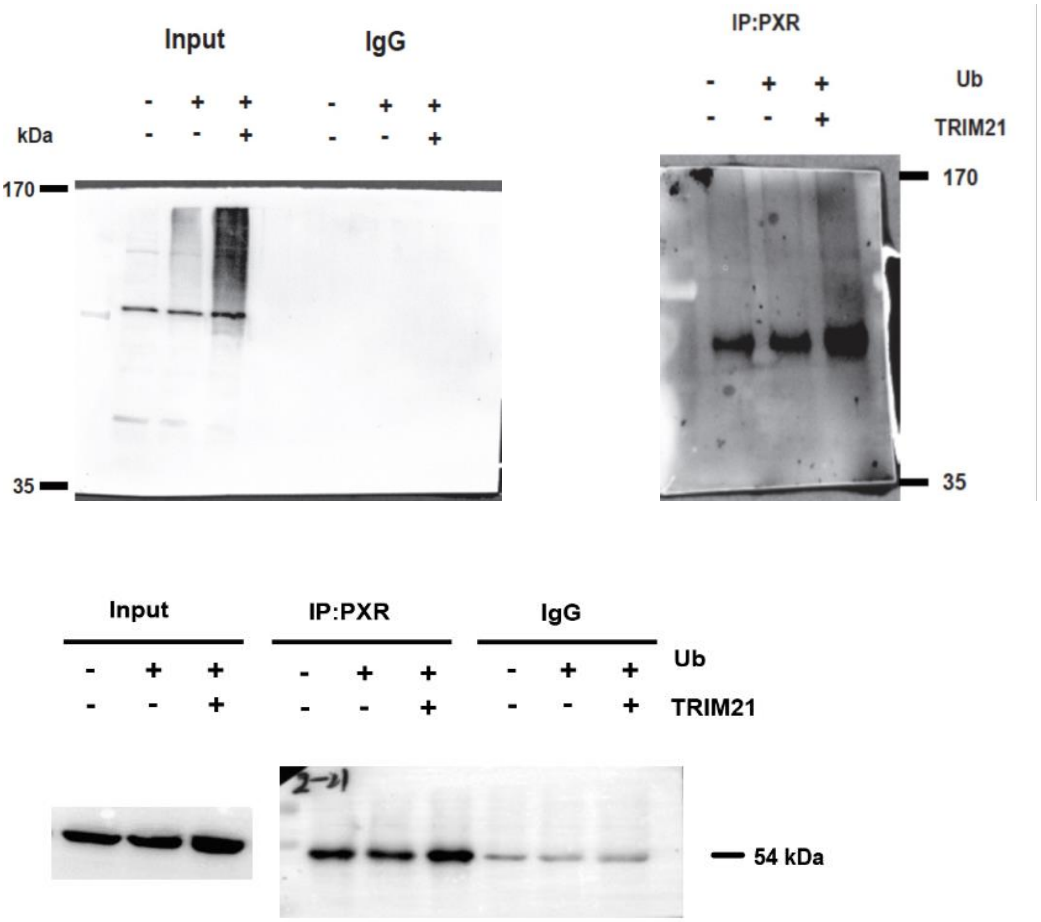
Input
IgG
kDa
170
35
IP:PXR
Ub
TRIM21
170
35
Input
IP:PXR
IgG
Ub
TRIM21
2-21
54 kDa

Figure 2

B

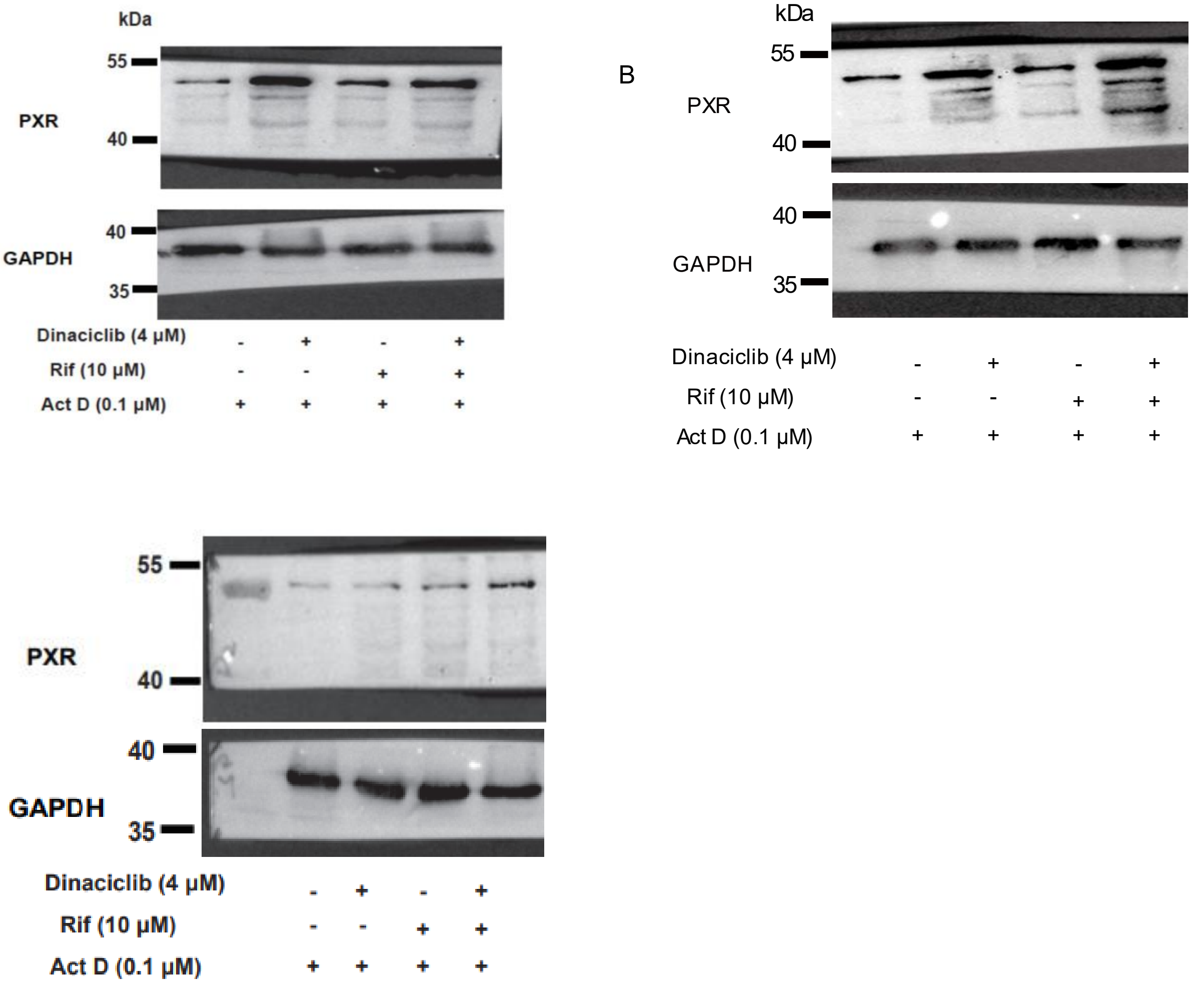
kDa
55
PXR
40
40
GAPDH
35
Dinaciclib (4 μM) - + - +
Rif (10 μM) - - + +
Act D (0.1 μM) + + + +
kDa
B
55
PXR
40
40
GAPDH
35
Dinaciclib (4 μM) - + - +
Rif (10 μM) - - + +
Act D (0.1 μM) + + + +
55
PXR
40
40
GAPDH
35
Dinaciclib (4 μM) - + - +
Rif (10 μM) - - + +
Act D (0.1 μM) + + + +

C

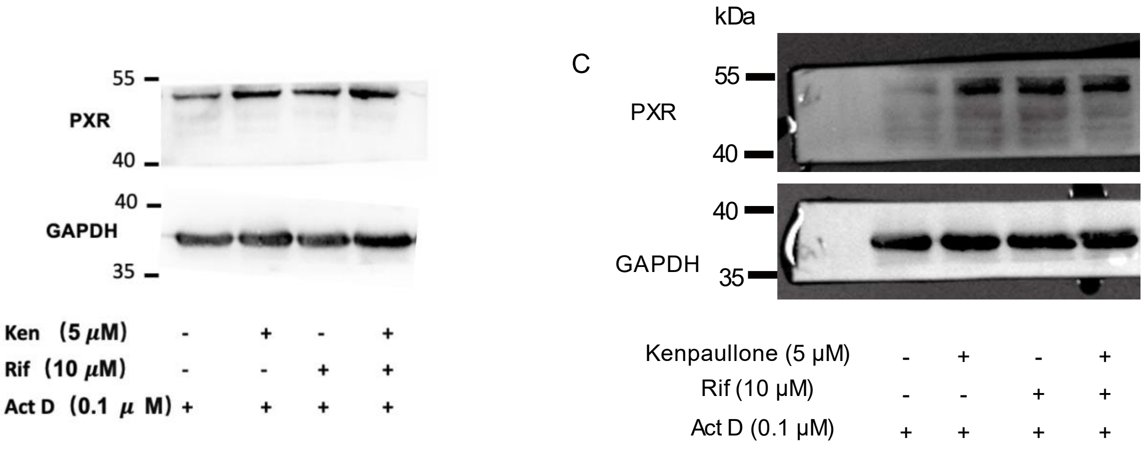
55
PXR
40
40
GAPDH
35
Ken (5 μM) - + - +
Rif (10 μM) - - + +
Act D (0.1 μ M) + + + +
kDa
C
55
PXR
40
40
GAPDH
35
Kenpaullone (5 μM) - + - +
Rif (10 μM) - - + +
Act D (0.1 μM) + + + +

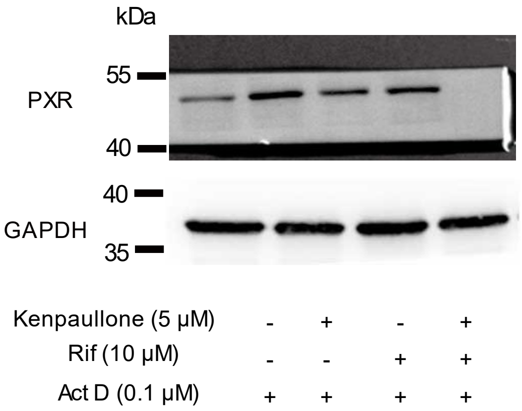
kDa
55
PXR
40
40
GAPDH
35
Kenpaullone (5 µM) - + - +
Rif (10 µM) - - + +
Act D (0.1 µM) + + + +

E

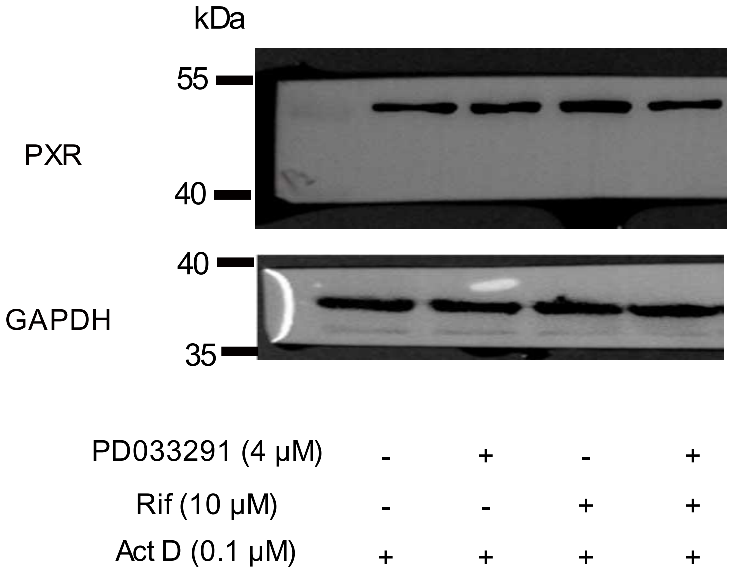
kDa
55
PXR
40
40
GAPDH
35
PD033291 (4 µM) - + - +
Rif (10 µM) - - + +
Act D (0.1 µM) + + + +

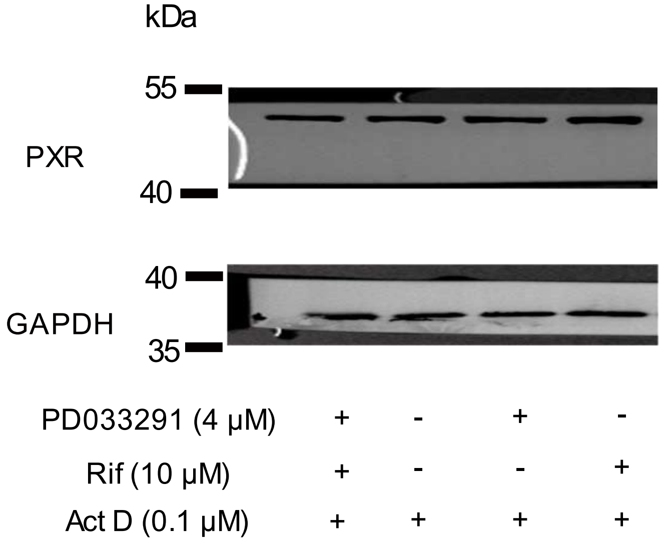
kDa
55
PXR
40
40
GAPDH
35
PD033291 (4 µM) + - + -
Rif (10 µM) + - - +
Act D (0.1 µM) + + + +

F

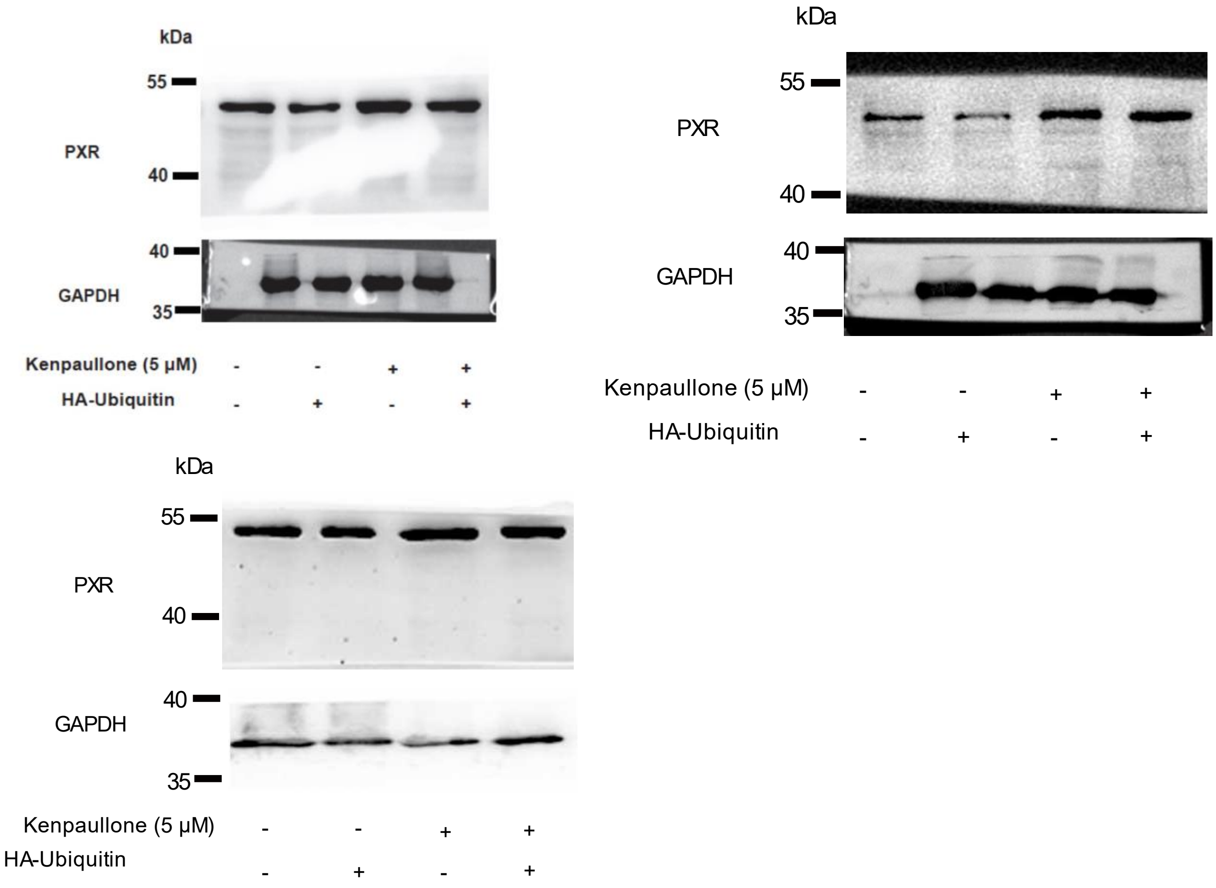
kDa
55
PXR
40
40
GAPDH
35
Kenpaullone (5 μM) - - + +
HA-Ubiquitin - + - +
kDa
55
PXR
40
40
GAPDH
35
Kenpaullone (5 μM) - - + +
HA-Ubiquitin - + - +
kDa
55
PXR
40
40
GAPDH
35
Kenpaullone (5 μM) - - + +
HA-Ubiquitin - + - +

Figure 3

A

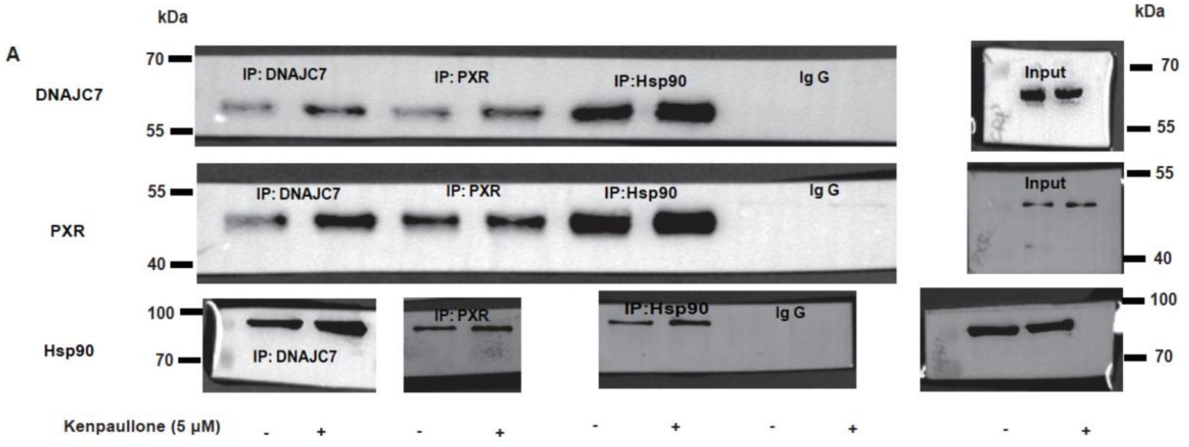

kDa
A
DNAJC7
PXR
Hsp90
IP: DNAJC7
IP: PXR
IP:Hsp90
Ig G
Input
70
55
40
100
Kenpaullone (5 µM)
- + - + - + - + - +

B

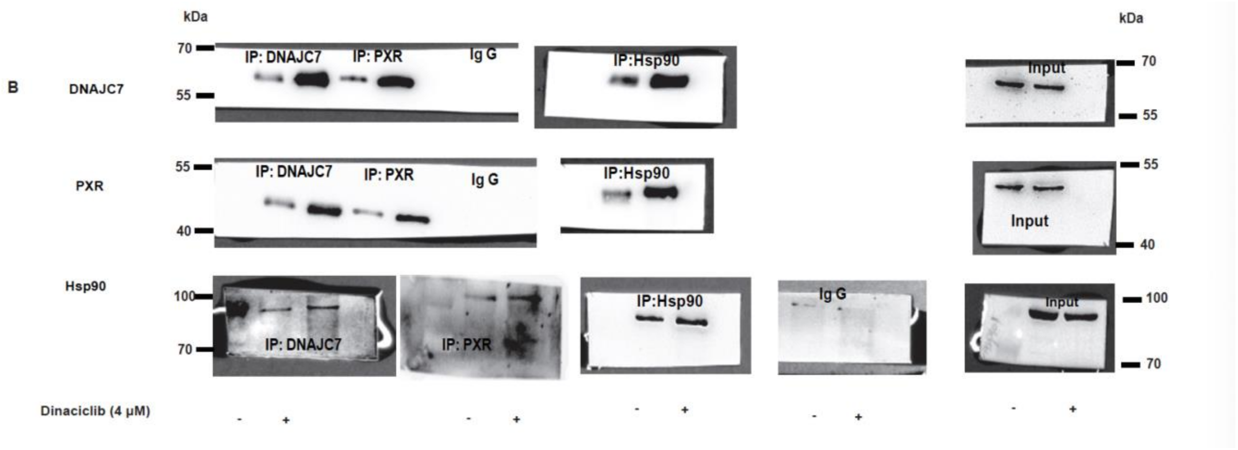

kDa
B
DNAJC7
PXR
Hsp90
IP: DNAJC7
IP: PXR
IP:Hsp90
Ig G
Input
70
55
40
100
Dinaciclib (4 µM)
- + - + - + - + - +

Figure 4

A

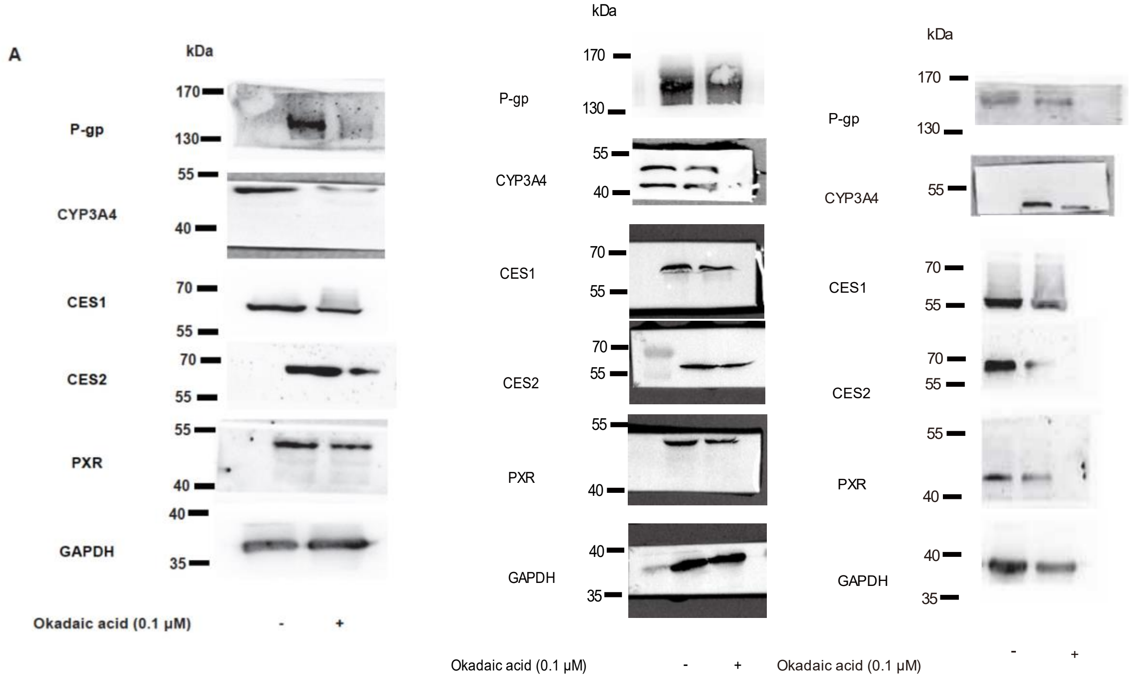
A
kDa
P-gp
CYP3A4
CES1
CES2
PXR
GAPDH
170
130
55
40
70
35
Okadaic acid (0.1 μM)
-
+
Okadaic acid (0.1 μM)
-
+
Okadaic acid (0.1 μM)
-
+

C

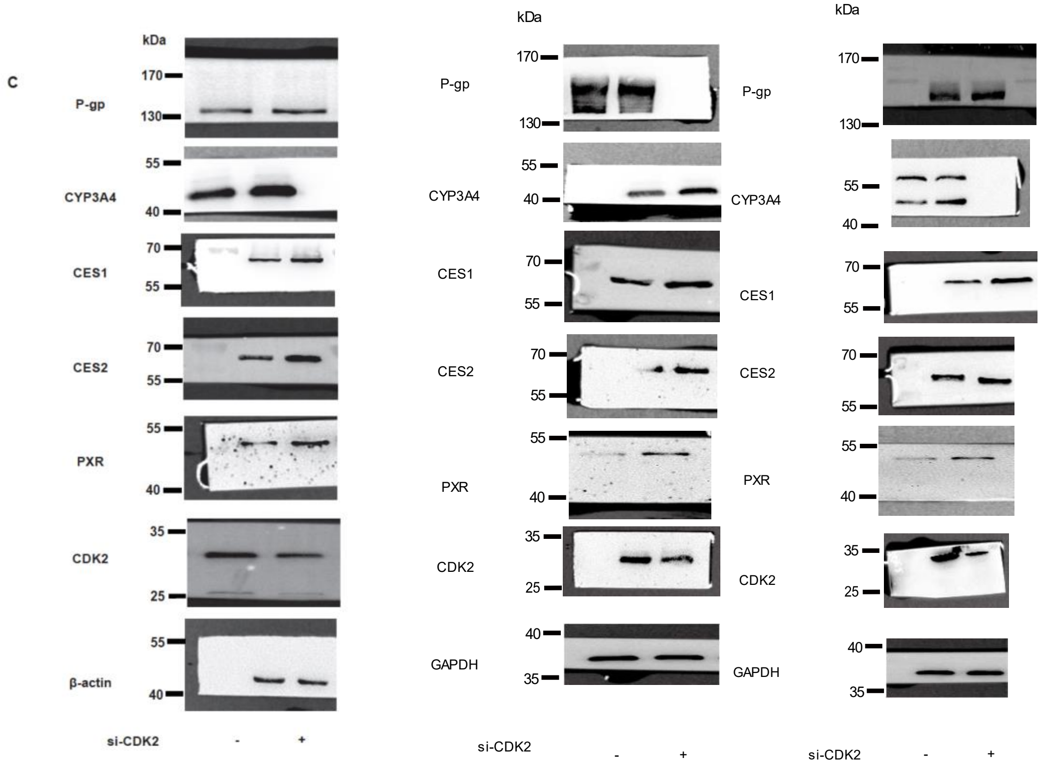
C
kDa
P-gp
CYP3A4
CES1
CES2
PXR
CDK2
β-actin
GAPDH
170
130
55
40
70
35
25
si-CDK2
-
+
si-CDK2
-
+
si-CDK2
-
+

D

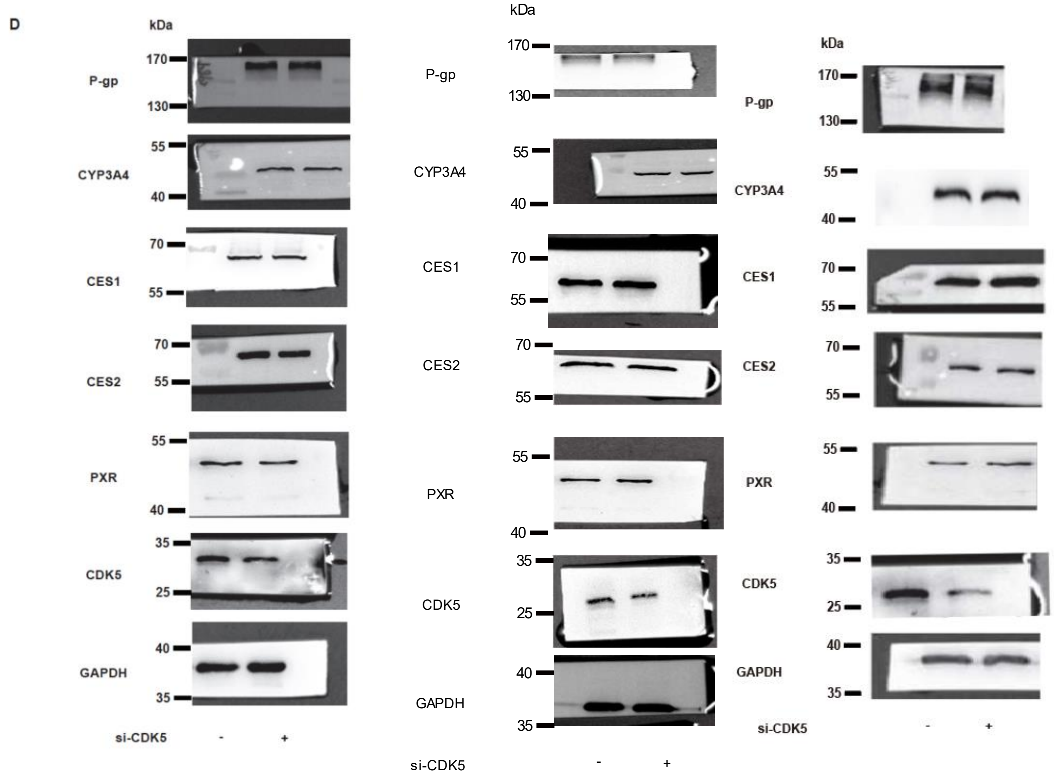
D
kDa
P-gp
170
130
CYP3A4
55
40
CES1
70
55
CES2
PXR
CDK5
35
25
GAPDH
si-CDK5
-
+

E

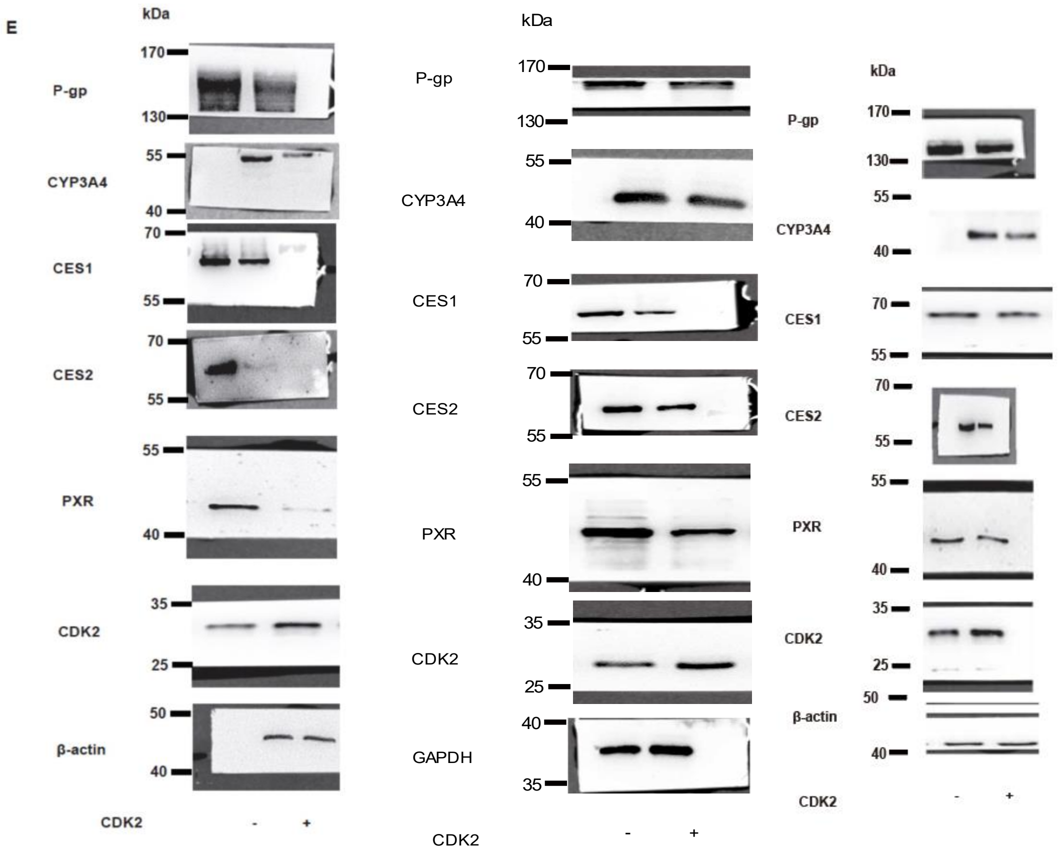
E
kDa
P-gp
170
130
CYP3A4
55
40
CES1
70
CES2
PXR
CDK2
35
25
β-actin
50
GAPDH
CDK2
-
+

Figure 6

A

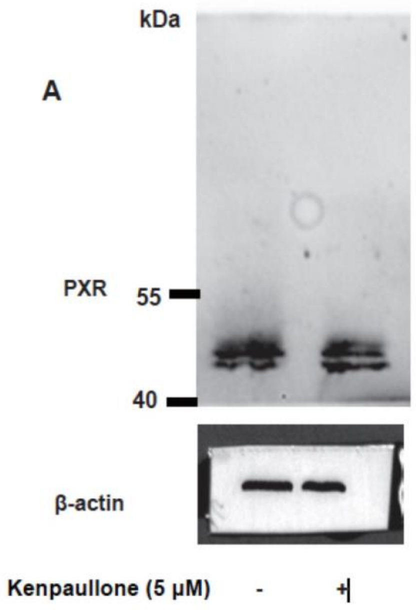
kDa
A
PXR
55
40
β-actin
Kenpaullone (5 μM)
-
+

B

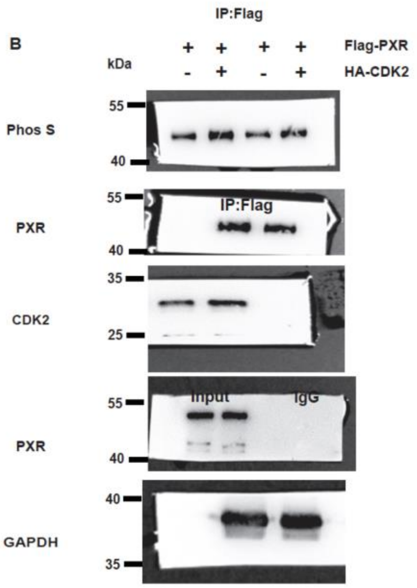
IP:Flag
B
Flag-PXR
HA-CDK2
kDa
Phos S
PXR
IP:Flag
CDK2
Input
IgG
PXR
GAPDH
55
40
35
25

C

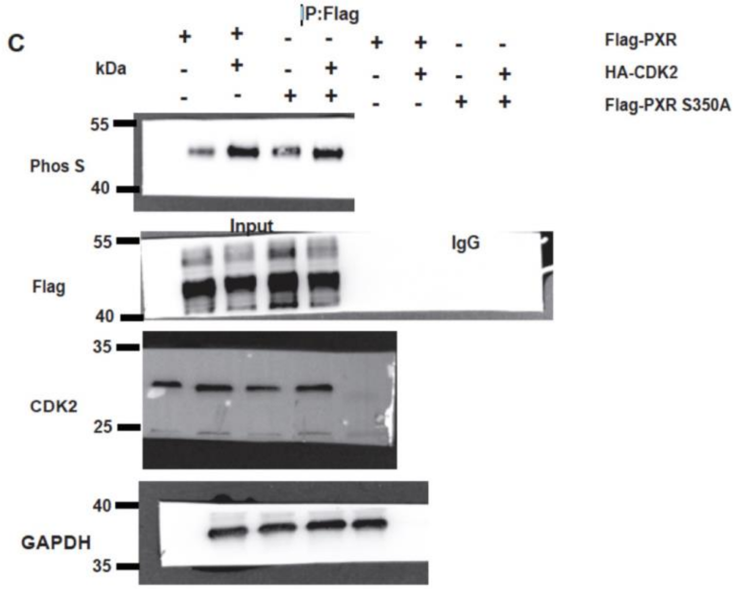
IP:Flag
C
kDa
Flag-PXR
HA-CDK2
Flag-PXR S350A
55
40
Phos S
Input
IgG
55
Flag
40
35
CDK2
25
40
GAPDH
35

D

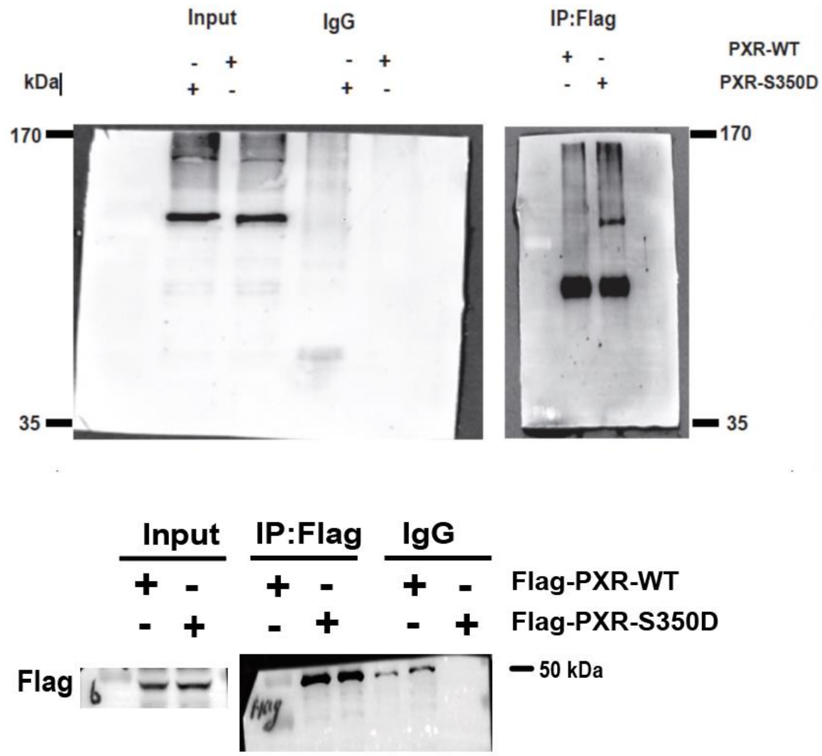
Input
IgG
IP:Flag
PXR-WT
kDa
PXR-S350D
170
35
170
35
Input
IP:Flag
IgG
Flag-PXR-WT
Flag-PXR-S350D
Flag
50 kDa
